# Enhancing life jacket detection for maritime search and rescue using YOLO models and illumination-robust preprocessing techniques

**DOI:** 10.1038/s41598-026-55717-0

**Published:** 2026-06-08

**Authors:** Jun Kit Kwong, Ban-Hoe Kwan, Humaira Nisar, Tian Swee Tan, Yan Chai Hum

**Affiliations:** 1 Department of Mechatronics and Biomedical Engineering, Lee Kong Chian Faculty of Engineering and Science, Universiti Tunku Abdul Rahman, Jalan Sungai Long, Bandar Sungai Long, Cheras, 43000 Kajang, Selangor Malaysia; 2 Department of Electronic Engineering, Faculty of Engineering and Green Technology, Universiti Tunku Abdul Rahman, Jalan Universiti, Bandar Barat, 31900 Kampar, Perak Malaysia; 3 Department of Biomedical Engineering & Health Sciences, Faculty of Electrical Engineering, Universiti Teknologi Malaysia (UTM), 81310 Johor Bahru, Johor Malaysia

**Keywords:** Computer vision, YOLO, Machine learning, Search and rescue operations, Safety, Object detection, Engineering, Mathematics and computing

## Abstract

Maritime Search and Rescue (SAR) operations are often challenged by vast search zones, poor visibility, and extreme lighting conditions, especially during nighttime missions. This study investigates the use of computer vision and object detection algorithms to automate life jacket detection and improve SAR effectiveness. To address the absence of domain-specific datasets, a custom image dataset featuring multiple life jacket types was developed. A two-fold methodology was adopted: evaluating the performance of YOLO object detection models (versions 5 through 12) on the dataset, and incorporating advanced image preprocessing techniques to enhance detection under challenging lighting conditions. The results demonstrate that preprocessing significantly improves detection performance in both overexposed and underexposed scenarios. Among all evaluated models, YOLOv10 achieved the strongest combination of precision and real-time inference speed (43.9 FPS on Tesla T4 GPU), making it a promising candidate for time-sensitive rescue applications. While individual cells of Tables 5, 6, 7, 8 and 9 show other detectors achieving higher precision under specific lighting × preprocessing combinations, YOLOv10 offers the best aggregate trade-off across the evaluated criteria. This work contributes a scalable benchmark solution for improving SAR outcomes by enabling faster and more reliable identification of individuals in distress at sea.

**Supplementary Information:**

The online version contains supplementary material available at 10.1038/s41598-026-55717-0.

## Introduction

**Fig. 1:**
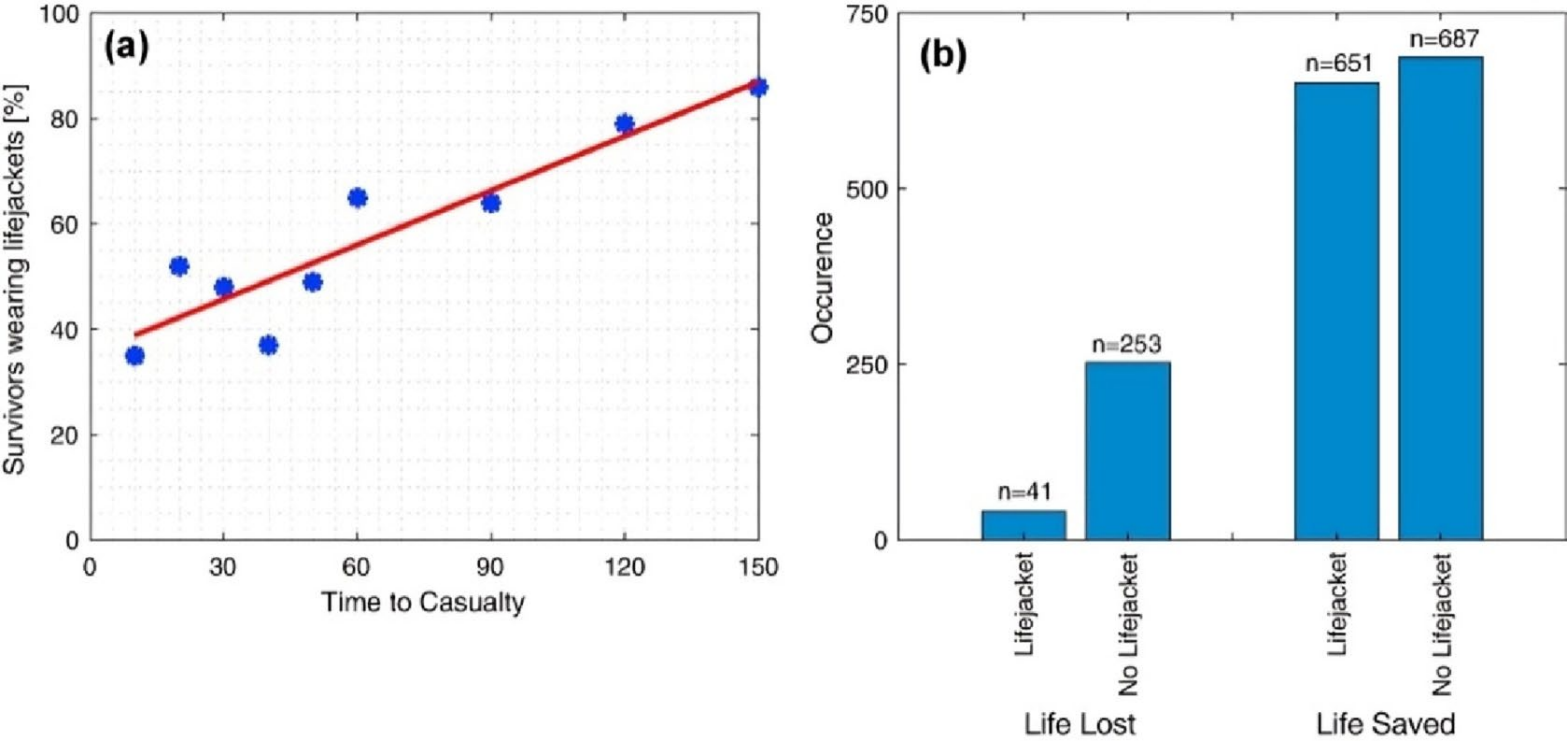
Comparison of survival rates in maritime incidents with and without life jackets, illustrating the critical role of flotation devices in reducing fatalities at sea^5^.

The vast expanse of the ocean holds both beauty and peril, and for those in distress at sea, every second counts. Early detection of individuals — particularly those wearing life jackets — is paramount to successful Search and Rescue (SAR) operations^1,2^. Detection delays carry severe consequences: hypothermia and drowning risks rise rapidly with time in the water, and traditional visual search methods struggle against vast search areas, rough seas, fog, and limited human vision at night^3,4,6–9^. As shown in Fig. 1, survival rate is closely tied both to life jacket use and to time-to-rescue^5^. Faster and more reliable detection therefore translates directly into improved survival outcomes.

Computer vision offers a promising path to faster detection. Object detection algorithms — particularly the YOLO (You Only Look Once) family — are well suited to recognising life jackets in images and video feeds from search vessels or aircraft, and have been used successfully in related maritime detection tasks^10–17^. However, real-world maritime environments present three classes of challenge that can degrade detection performance: variable lighting (from bright sunlight to darkness)^18–22^, wave disturbances and surface spray^23–25^, and partial occlusion of subjects by water, debris, or other objects^26–29^. Among these, illumination variability is the most pervasive factor across SAR missions and is the focus of the present work.

Deep learning has driven state-of-the-art results across a wide range of vision applications — including medical image anlaysis[30], autonomous-vehicle control[31], business and operational analytics[32], and face recognition[33,34] — and the same data-driven paradigm now extends to image preprocessing. To mitigate illumination effects, image preprocessing techniques — both classical (histogram equalisation, CLAHE, gamma correction) and deep-learning-based (exposure correction, low-light image enhancement) — have been proposed, with deep-learning methods generally outperforming classical ones on benchmarks^35–44^. To our knowledge, however, no prior study has systematically evaluated the combined effect of modern YOLO detectors and modern preprocessing pipelines on a curated life jacket dataset under controlled lighting variations.

This study makes four contributions. First, we curate a custom life jacket detection dataset of 1,630 images covering four life jacket types, addressing the absence of publicly available data for fine-grained life jacket classification. Second, we benchmark eight YOLO versions (v5–v12) alongside two non-YOLO baselines (SSD MobileNet V2 and Faster R-CNN Inception-ResNet-v2) on this dataset. Third, we evaluate twelve preprocessing methods (two classical, ten deep-learning-based) under five simulated lighting conditions to identify which methods are most effective for which lighting failure mode. Fourth, we provide an interpretative analysis of the detector × preprocessing × lighting interaction that yields actionable recommendations for SAR-relevant deployment, while transparently identifying the gap between this controlled benchmark and real maritime operating conditions.

## Background

This section provides a brief overview of relevant research in computer vision object detection, particularly focusing on challenges specific to maritime environments. We delve into computer vision techniques, image enhancement strategies, and their integration to improve object detection accuracy in marine contexts. This review will lay the groundwork for the approach proposed in this article.

### Related work

Life jacket detection sits at the intersection of two well-developed but largely separate literatures: general maritime object detection, and life-jacket-relevant aerial detection. We summarise each, then identify the specific gap addressed by the present work.

#### General maritime object detection

Early approaches relied on traditional computer vision — colour segmentation and edge detection — and struggled with lighting variation, occlusion, and complex maritime backgrounds^56–60^. Deep learning, particularly CNN-based detectors, has progressively closed these gaps; multiple studies have demonstrated improved accuracy and robustness on maritime targets such as ships, buoys, and people in distress^14,21,46,47,48,61–63^. Within this thread, YOLO and its variants are the dominant choice for real-time maritime applications^10,12,49,50,51,52–55,64,65^, with several works adapting the architecture (k-means anchor optimisation, lightweight backbones, attention modules) to specific maritime scenarios.

#### Life-jacket-relevant aerial detection

A smaller body of work has targeted life jackets directly, predominantly in UAV-based settings. Cafarelli et al.^15^ introduced MOBDrone, an aerial dataset for person-overboard detection, while Tallón-Ballesteros & Beltrán-Barba^17^ developed YoloOW for UAV-based search and rescue with explicit handling of variable object sizes and water reflections. Du et al.^21^ released the SeaDronesSee benchmark with metadata-aware annotations across altitudes and viewing angles. These works establish the feasibility of aerial life-jacket detection but share two limitations relevant to broader SAR deployment: they assume aerial viewpoint exclusively, and they evaluate on imagery captured under broadly favourable lighting.

#### Gap addressed

Despite this progress, three issues remain underexplored: (i) systematic evaluation across multiple life jacket types rather than a single binary “person-in-water” class; (ii) controlled assessment of preprocessing-detector interaction under explicit lighting failure modes (overexposure, underexposure); and (iii) cross-platform applicability beyond high-altitude UAV imagery. The present work addresses all three by benchmarking eight YOLO versions and two non-YOLO baselines against a four-class life jacket dataset, with twelve preprocessing methods evaluated under five lighting conditions on a fixed-viewpoint test set.

### Computer vision techniques for object detection

Computer vision has emerged as a potent tool for object detection and recognition in diverse fields, including maritime applications. Object detection algorithms, which analyse visual data to identify and locate objects within images or videos, have been extensively researched and applied to tasks like autonomous driving, surveillance, and medical image analysis. Computer vision techniques can be categorized into sensor-based and vision-based approaches.

Sensor-Based Techniques rely on specialized sensors to gather data beyond the visible spectrum. It offers the advantage of capturing data beyond human perception, such as infrared heat signatures^68–70^ or precise distance measurements from lidar^71–74^. These techniques are often more robust to lighting conditions and can provide accurate and precise measurements. For instance, Zhang et al.^63^ have developed a novel Self-Powered Life Jacket (SPLJ) that employs a wireless body area network (WBAN) and deep learning analytics to monitor the wearer’s underwater movements and condition. The SPLJ utilizes triboelectric fibre sensors to capture human movement data, which is then transmitted wirelessly to a central system for analysis. By analysing the movement patterns, the system can accurately detect drowning incidents and trigger appropriate rescue actions. This technology has the potential to significantly improve survival rates in water-related accidents and has broader applications in various fields, including swimming instruction, rescue operations, and underwater exploration. Park et al.^75^ contributes to the advancement of maritime safety by providing a reliable method for detecting small objects in aerial imagery. This technology can be used to improve search and rescue operations, environmental monitoring, and other maritime applications. Wang et al.^76^ has developed an intelligent life jacket system to enhance water safety. This system incorporates a microcontroller, water sensor, GPS module, and wireless communication capabilities to detect falls into water, transmit location data, and trigger alarms. The system has been tested and found to be effective in detecting falls, accurately locating the wearer, and sending timely alerts. Nguyen et al.^77^ leverages IoT technology to enhance water safety at tourist sites. By equipping life jackets with sensors and wireless connectivity, the system can track wearers’ locations and conditions in real-time. In case of emergencies, automated alerts are sent to rescue teams, enabling timely intervention. Additionally, the collected data can be analysed to identify trends and implement preventive measures, improving overall water safety. Serra et al.^78^ proposes a new approach to emergency communication for survivors of maritime accidents. By integrating two dipole antennas into a life jacket, the system can establish satellite communication, allowing for distress signals to be sent even in remote locations. The antennas are designed to be efficient and reliable, even when worn by a person. The system offers potential benefits for maritime safety by providing a reliable means of communication for survivors. However, sensor-based techniques were more expensive due to specialized hardware and may have limitations in range or field of view. Additionally, integrating and processing data from multiple sensors can be complex and computationally intensive, and sensors can be susceptible to interference from external factors.

Vision-based techniques primarily utilize cameras to capture visual information such as images or videos. Examples of these techniques include image processing^79–82^, object detection^57,83–85^, object tracking^86–89^, and facial recognition^90–93^. They offer the advantages of being low-cost and non-invasive while capturing a wealth of visual information. These techniques are versatile and applicable to a wide range of tasks. For instance, Kiefer et al.^94^ proposes a new approach to video object detection and tracking on UAVs by utilizing metadata. By incorporating information about the real-world coordinates of objects, the system can improve the accuracy and robustness of various computer vision tasks, including object detection, tracking, and anomaly detection. This approach offers a more interpretable and reliable representation of object locations, leading to better performance. Cafarelli et al.^15^ introduces a new dataset called MOBDrone, specifically designed for detecting people in distress at sea in aerial imagery. This dataset contains over 125,000 images with more than 180,000 annotated objects, including people overboard. The scholars have conducted a thorough evaluation of several state-of-the-art object detectors on this dataset, highlighting the challenges of detecting small and distant objects in maritime environments. Zhao and Song^14^ proposes a novel method to optimize the YOLOv8 model for UAV-based maritime rescue object detection. This optimization focuses on improving both accuracy and real-time performance. Key contributions include a modular plug-and-play approach that allows for flexible and easy implementation of various optimization techniques. Experiments on the SeaDronesSee dataset demonstrate significant improvements in both accuracy and speed compared to the baseline YOLOv8 model. Ye and Li^95^ proposes an improved U-Net model for automatic life jacket detection in images. Key improvements include replacing max-pooling with a special convolutional layer, adding a dropout layer, and using reflection padding. These modifications aim to extract more features and prevent overfitting. The model achieves an accuracy of approximately 93% and an IoU of 4% higher than the original U-Net, demonstrating its effectiveness in accurately identifying life jackets. Huang and Chong^27^ proposes a novel approach to multi-object tracking in maritime UAV and USV scenarios. The key innovation lies in its unsupervised learning approach, which eliminates the need for expensive video annotation data. By leveraging self-supervised learning on ImageNet, the system learns to represent instances effectively. By combined with high-quality object detectors, enables simple and efficient multi-object tracking. The proposed method has demonstrated state-of-the-art performance on various benchmarks, including BDD100K MOT, MOTS, and Waymo 2D MOT. However, all vision-based techniques are susceptible to varying lighting conditions and can be computationally intensive. Additionally, occlusions and limited depth perception can hinder accurate object detection and tracking.

The state-of-the-art vision-based object detection techniques have seen the emergence of several powerful algorithms, each with its unique strengths and weaknesses. Among the most prominent are YOLO (You Only Look Once), Faster R-CNN (Region-based Convolutional Neural Network), and SSD (Single Shot MultiBox Detector).

YOLO^11^ single-stage detection approach, where it predicts bounding boxes and class probabilities directly from the input image, contributes to its impressive speed. This makes it particularly well-suited for real-time applications like autonomous vehicles^96,97^, surveillance systems^98,99^ and so on. However, YOLO reliance on a fixed number of bounding box predictions can sometimes lead to difficulties in accurately detecting small or closely grouped objects.

Faster R-CNN^100^ two-stage approach, involving region proposal generation followed by object classification and bounding box regression, allows it to achieve higher accuracy than YOLO, especially in complex scenes with numerous objects. This makes it a popular choice for applications demanding high precision, such as medical image analysis^101,102^, satellite imagery interpretation^103,104^ and others. Nevertheless, the two-stage nature of Faster R-CNN can increase its computational cost, potentially limiting its real-time performance, especially on resource-constrained devices.

SSD^105^ offers a compromise between YOLO’s speed and Faster R-CNN’s accuracy. Its single-shot approach, utilizing multiple feature maps at different scales, enables it to efficiently detect objects of various sizes. This makes SSD suitable for a wide range of applications, including real-time object tracking^106,107^ and video analytics^108,109^. However, SSD’s reliance on default bounding boxes can sometimes lead to suboptimal localization, particularly for objects that deviate significantly from the predefined shapes. Additionally, SSD may struggle to accurately detect objects with significant occlusions or complex backgrounds.

The selection of object detection algorithms for life jacket detection is crucial, as it directly impacts the system’s performance. Given the need for real-time processing and the potential complexity of maritime environments, YOLO is a strong candidate. To optimize performance, we will evaluate various YOLO versions, from v5 to v12, considering factors such as accuracy, speed, and computational cost. The goal is to identify the most suitable YOLO version for our specific application, balancing real-time requirements with the need for reliable and accurate life jacket detection.

### Challenges specific to maritime environments

The maritime environment presents unique challenges for computer vision-based object detection, particularly in the context of life jacket detection. These challenges stem from factors such as varying lighting conditions, wave disturbances, occlusions and others.

One of the primary challenges in maritime object detection is the dynamic nature of lighting conditions at sea. Fluctuations in sunlight intensity, atmospheric conditions, and water reflections can drastically affect image quality. Extreme deviations from optimal exposure, such as overexposure and underexposure, can significantly degrade the performance of object detection models. To address these issues, researchers have explored various techniques, including image enhancement and specialized deep learning architectures. Image enhancement techniques like histogram equalization^110,111,112,113^^114^, level adjustment, and contrast enhancement^115–117^ can improve image quality in both overexposed and underexposed conditions. However, for more complex scenarios, there will be more specialized image enhancement algorithms and architectures for overexposed and underexposed conditions^118–120^. These techniques effectively enhance visibility by increasing or reducing brightness as needed and reducing noise, thus facilitating information extraction from challenging lighting conditions. Additionally, adaptive thresholding techniques^121^ can be employed to segment objects from the background, even in overexposed or underexposed images, further enhancing image analysis and understanding.

Wave disturbances and surface spray, coupled with the dynamic nature of the marine environment, pose significant challenges to maritime object detection systems. Wave-induced motion can blur images, making it difficult to accurately identify and localize objects. Additionally, surface spray can obscure objects, particularly those near the water’s surface, further hindering detection efforts. To alleviate these problems, researchers have explored various strategies. High-Definition Restoration (HDR) technology^122,123^ and motion compensation techniques like frame stabilization^124,125^, can help to reduce the negative effects of wave-induced motion on image quality. For instances^126^, proposed an EL-YOLO based on YOLOv8, a novel object detection algorithm specifically designed for maritime scenarios. By incorporating AWIoU for improved bounding box regression, SMFN for enhanced feature extraction, and GDFP for model streamlining, EL-YOLO achieves significant improvements in both accuracy and efficiency. Experimental results demonstrate its superior performance in detecting maritime objects, even in challenging conditions like low-quality images, occlusions, reflections and wave disturbances. Chaudhuri et al.^45^ presented a novel statistical approach for detecting dark curvilinear features in SAR images, which are indicative of ocean disturbances like ship wakes, submarine wakes, or oil spills. The method involves several steps: image enhancement, iterative segmentation, hole filling, thinning, and feature linking. The proposed approach is evaluated on both synthetic and real-world SAR images, demonstrating superior accuracy and efficiency compared to existing techniques.

Occlusions, where objects are partially or fully hidden by other objects, present another significant hurdle. In the context of life jacket detection, occlusions can occur due to waves, debris, or other objects in the water. To address this challenge, researchers have explored techniques such as contextual information^127,128^, part-based detection^129–131^, and instance segmentation^132,133^. For instances^134^, introduced a novel object detection model called YOLO-ACN, which aims to improve detection accuracy and speed, especially for small and occluded objects. The model incorporates several improvements, including an attention mechanism to focus on small targets, CIoU loss for more accurate bounding box regression, optimized Soft-NMS for better object filtering, and depth wise separable convolutions for faster inference. Experimental results on the MS COCO and KAIST datasets demonstrate that YOLO-ACN outperforms state-of-the-art models in terms of both accuracy and speed, particularly for small object detection and occluded objects. Wang et al.^135^ introduced a novel approach to detect partially occluded objects using CompositionalNets. By segmenting the context from the object during training, the model learns a more robust representation of the object itself. Additionally, a robust bounding box voting scheme is employed to accurately estimate bounding boxes for partially occluded objects. Experimental results demonstrate significant improvements in detection accuracy, especially in challenging occlusion scenarios. Xiao et al.^136^ introduced TDAPNet, a novel approach to enhance the robustness of deep neural networks under occlusion. By integrating prototype learning, partial matching with top-down attention, and feedback mechanisms, the model effectively addresses the challenges posed by occlusions. This approach enables the model to learn compact data representations, focus on informative image regions, and mitigate the negative impact of occlusions during feature extraction. Experimental results demonstrate significant improvements in classification accuracy, particularly on datasets with partially occluded objects, making the model more robust against occlusion.

While maritime object detection faces numerous challenges, including varying weather conditions, wave disturbances, and occlusions, this research will primarily focus on addressing the issue of dynamic lighting conditions. By developing advanced image enhancement techniques and robust detection algorithms, we aim to significantly improve the accuracy and reliability of maritime object detection systems, ultimately enhancing safety and efficiency in maritime operations.

### Integration of image enhancement techniques

Image enhancement methods fall into two technical families. Classical methods — histogram equalisation^110,112^, gamma correction^137,138^, curve mapping^139^, CLAHE^140^, and Retinex-based decomposition^141–146^— apply predefined intensity transformations to redistribute pixel values. They are computationally cheap and well understood, but typically struggle with images containing both overexposed and underexposed regions, and with non-uniform illumination. Deep-learning methods learn the transformation directly from data, allowing context-dependent correction. Notable architectures include Retinex-inspired decomposition networks^148,150^, paired-data exposure correction^43,147,149^, unsupervised low-light enhancement^44,159,160^, and transformer-based approaches that handle ultra-high-resolution input^151,152,162^. State-of-the-art methods in this family — including Afifi et al.‘s coarse-to-fine exposure correction^43^, Cui et al.‘s lightweight transformer^158^, and Zamir et al.‘s MIRNet-v2^153^— achieve top performance on standard benchmarks but vary substantially in their behaviour under specific lighting failure modes, motivating the systematic comparison conducted in the section “Evaluation protocol”. The integration of preprocessing with downstream detection or recognition pipelines has also been demonstrated in non-maritime domains such as face recognition^154^, underwater object detection^103,155^, network intrusion detection^156^, and medical imaging^157^, indicating the breadth of applications in which preprocessing-detector coupling has been shown to improve robustness.

For the present study, we evaluated twelve preprocessing methods drawn from both families: two classical (histogram equalisation, CLAHE) and ten deep-learning-based (Afifi et al.^43^, Cui et al.^158^, H. Nguyen et al./PSENet^152^, H. Wang et al./LCDPNet^151^, Nsampi et al.^161^, T. Wang et al.^162^, Bai et al./RetinexMamba^159^, C. Guo et al./Zero-DCE^160^, and the contrast-enhancement and lowlight-enhancement variants of Zamir et al./MIRNet-v2^153^. All deep-learning methods were applied using the authors’ publicly released pretrained weights without retraining or fine-tuning, ensuring that comparisons reflect each method’s intended generalisation behaviour. The full implementation details and rationale are reported in the section “Image preprocessing methods”.

### Research overview

This research builds upon existing literature by combining the most advanced YOLO architectures with image enhancement techniques. Focusing on life jacket detection, a critical application in maritime search and rescue and one of the preventive measures, this research aims to contribute to the development of more robust and effective systems.

- Leveraging Different YOLO Versions: This research benefits from advancements in the YOLO family of object detection algorithms. By utilizing versions from YOLOv5 to YOLOv12, we aim to capitalize on their improved performance and efficiency. These algorithms have demonstrated state-of-the-art results in various object detection tasks, making them suitable for the challenging task of life jacket detection in maritime environments.
- Combining Object Detection and Image Enhancement: This research proposes a two-pronged approach that integrates object detection with image enhancement techniques. This combination addresses the limitations posed by maritime environments, such as varying lighting conditions. By enhancing image quality, we aim to improve the accuracy and reliability of life jacket detection, even under challenging conditions.
- Focusing on Life Jacket Detection: The research specifically targets the detection of life jackets, a critical application in maritime search and rescue. By covering four different types of life jackets, this research aims to ensure the system’s effectiveness in detecting a wide range of life jacket designs commonly used at sea. This focus on life jacket detection contributes to the development of more specialized and efficient systems for maritime safety.

By addressing the limitations of previous studies and incorporating the latest advancements in computer vision, this research aims to contribute to the development of more robust and effective life jacket detection systems. These advancements have the potential to significantly improve the efficiency and success rate of search and rescue operations, ultimately saving lives at sea.

## Methodology

This study evaluates eight YOLO versions (YOLOv5 through YOLOv12) for life jacket detection in maritime search and rescue (SAR) contexts, with SSD MobileNet V2 and Faster R-CNN Inception-ResNet-v2 as baseline detectors. The pipeline comprises four stages: (i) construction of a custom four-class life jacket dataset, (ii) training of all ten detectors under matched compute budgets, (iii) augmentation of the test set under five lighting conditions, and (iv) evaluation with twelve image preprocessing methods (two traditional and ten deep-learning-based). All experiments were conducted on Google Colab’s free-tier Tesla T4 GPU under Python 3.10.12, PyTorch 2.3.1, and TensorFlow 2.15.1. The complete workflow is illustrated in Fig. 4.

### Custom life jacket dataset

#### Image collection

**Fig. 2:**
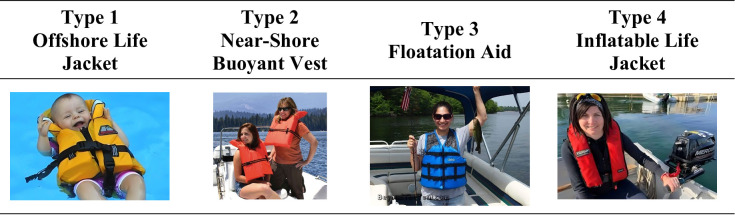
Types of life jackets in the dataset. Sample images are adapted from the “Life Jacket” dataset by CONTIL, available from Roboflow Universe at https://universe.roboflow.com/contil-rn9fi/life-jacket-kt521 and licensed under CC BY 4.0 (https://creativecommons.org/licenses/by/4.0/). Images were cropped, resized, and enhanced for presentation quality.

A dataset of 1,630 images covering four life jacket types — Type 1 (Offshore Life Jacket), Type 2 (Near-Shore Buoyant Vest), Type 3 (Floatation Aid), and Type 4 (Inflatable Life Jacket) — was constructed between August and October 2022. Because no publicly available dataset for fine-grained life jacket classification existed at the time, images were collected through web scraping. A custom Python script using the `selenium` and `requests` libraries was used to download candidate images from Google Images and Bing Images search results. Search queries combined the four life jacket type names (e.g., “offshore life jacket”, “inflatable life jacket”, “near-shore buoyant vest”, “floatation aid”) with contextual terms such as “person wearing”, “boat”, “marine”, and “maritime rescue” to maximise visual diversity across viewpoints, scenes, and life jacket appearances. The four life jacket types are illustrated in Fig. 2.

#### Inclusion, exclusion, and deduplication

After scraping, all candidate images were manually reviewed by the first author. Images were retained if they clearly depicted at least one life jacket of one of the four target types in a recognisable form. Images were excluded if (i) the life jacket type was ambiguous or could not be confidently classified, (ii) the image was a stock product photograph without environmental context (e.g., catalogue images on plain white backgrounds), or (iii) the image quality was insufficient for reliable annotation due to severe blur, very small object size, or heavy watermarking. Visual near-duplicates were also removed manually during this review pass. The final 1,630 images represent unique scenes after this deduplication step.

#### Annotation

All 1,630 images were manually annotated by the first author using LabelImg, producing axis-aligned bounding boxes in YOLO format with one of the four class labels per box. Single-annotator labelling is acknowledged as a limitation (Sect. “Result discussion”); inter-annotator agreement was not measured.

#### Train, validation, and test partitioning

The dataset was partitioned into three disjoint subsets at the image level: 1,430 training images, 100 validation images, and 100 test images. To enable balanced per-class evaluation, the test set was constructed deterministically with exactly 25 images per life jacket class (4 classes × 25 images = 100). The remaining 1,530 images were then split randomly into training and validation sets (with a 195-image background subset retained only for training, to expose the detectors to negative examples without life jackets). The full per-class distribution is reported in Table 1. Because images were collected from independent web sources rather than from continuous video streams, the risk of source-level information leakage between splits is low.

**Table 1:** Class-wise distribution of images across training, validation, and test sets.

Class	Train	Val	Test	Total
Type 1 - Offshore Life Jacket	297	23	25	345
Type 2 - Near-Shore Buoyant Vest	312	20	25	357
Type 3 - Floatation Aid	326	29	25	380
Type 4 - Inflatable Life Jacket	300	28	25	353
Others (Background)	195	0	0	195
**Total**	1430	100	100	1630

#### Dataset limitations

The dataset is built exclusively from web-scraped imagery and therefore inherits the visual biases of public image search: it predominantly contains single, clearly visible life jackets in relatively uncluttered scenes, captured under daylight conditions and from fixed viewpoints. It does not include extreme weather (heavy fog, storms, high sea states), genuine nighttime imagery, multi-target scenes with overlapping subjects, very small or distant targets (objects smaller than 32 × 32 pixels in image space), or heavily occluded subjects. Operational implications of these limitations are discussed in the section “Result discussion”; expansion of the dataset along each of these dimensions is identified as a priority for future work.

### YOLO training procedure

All eight YOLO versions (v5, v6, v7, v8, v9, v10, v11, v12) were trained under an identical protocol to enable fair cross-version comparison. Training used the Ultralytics framework with the following hyperparameters: input resolution 320 × 320 pixels, batch size 5, 300 epochs, default optimiser settings (SGD with the Ultralytics default learning rate schedule). The “small” or “tiny” variant of each YOLO version (e.g., `yolov5s.yaml`, `yolov11s.yaml`) was used to maintain comparable parameter counts and to keep training feasible within the Tesla T4 memory budget. Training produced 1,430/5 = 286 gradient updates per epoch, giving 85,800 total gradient updates per model.

#### Initialisation from random weights

A key design choice was to train all YOLO versions from random initialisation rather than from COCO-pretrained weights (`pretrained=false` in the Ultralytics `yolo train` command). This decision was made deliberately to ensure that all eight YOLO versions started from identical initialisation conditions. COCO-pretrained checkpoints differ across YOLO versions in their training data scale, augmentation pipelines, and release dates; using version-specific pretrained weights would have introduced a confounding factor that varies systematically with the very dimension we wished to compare. Training from scratch eliminates this source of bias at the cost of lower absolute precision values than would be obtained with COCO fine-tuning. Readers comparing our reported precision values with those in the original YOLO papers should note that those papers report results after COCO pretraining followed by fine-tuning on a much larger benchmark (typically COCO itself, with ~ 118,000 training images).

#### Overfitting controls

Given the modest dataset size (1,430 training images), several measures were taken to mitigate overfitting:

- Random seed. The Ultralytics default seed (`seed = 0`) was used for reproducibility.
- Data augmentation. The default Ultralytics augmentation pipeline was applied during training: HSV colour jitter (h = 0.015, s = 0.7, v = 0.4), random translation (± 10%), random scale (± 50%), horizontal flip with probability 0.5, and Mosaic 4-image augmentation with probability 1.0.
- Validation monitoring. Validation metrics were computed every epoch by the Ultralytics framework, with model checkpoints saved every 100 epochs (`save_period = 100`). The final reported model for each YOLO version is the `best.pt` checkpoint — the checkpoint with the highest validation mAP@0.5 across the full 300-epoch training run, selected automatically by the framework.
- No early stopping. Training was allowed to run for the full 300 epochs without early stopping, which was a deliberate choice to expose all models to the same training budget. We verified post hoc by inspection of training and validation loss curves (provided in Supplementary Information) that validation loss did not diverge substantially from training loss in late epochs for any model, indicating that overfitting was not a dominant failure mode under this regime.

The complete training config for each YOLO model was at Table 2.

**Table 2:** Unified Training Configuration Applied to All YOLO Versions.

Parameter	Value	Description
task	“detect”	Specifies the task type as object detection
mode	“train”	Indicates training mode
imgsz	320	Input image size (320 × 320 pixels)
batch	5	Number of samples per training batch
epochs	300	Total number of training epochs
data	“dataset.yaml”	Path to dataset configuration file
model	“< yolo_config>.yaml”	Model configuration file (varies by YOLO version)
name	“< version_name>”	Experiment name specific to each YOLO version
project	“Trained/< version>”	Output directory for saving model checkpoints and logs
save_period	100	Frequency (in epochs) to save model checkpoints

We acknowledge as a limitation that, given the dataset size, k-fold cross-validation would have provided more robust estimates than the single train/validation/test hold-out we used. Single-fold evaluation was a pragmatic choice driven by Colab compute constraints; we partially compensate for this through bootstrap test-set confidence intervals (Sect. “Image preprocessing methods”).

### Baseline detector configurations (SSD and faster R-CNN)

Two non-YOLO detectors were included as baselines to provide architectural diversity in the comparison:

- SSD MobileNet V2 (`ssd_mobilenet_v2_320 × 320_coco17_tpu-8`): a single-shot detector with a MobileNet V2 backbone, representative of efficient single-stage architectures.
- Faster R-CNN Inception-ResNet-v2 (`faster_rcnn_inception_resnet_v2`): a two-stage detector with an Inception-ResNet-v2 backbone, representative of high-accuracy two-stage architectures.

Both baselines were obtained from the TensorFlow 2 Object Detection Model Zoo and trained using the `model_main_tf2.py` training script. Unlike the YOLO models, the two baselines were initialised from their respective COCO-pretrained checkpoints (`fine_tune_checkpoint_type: “detection"`); training the much larger Inception-ResNet-v2 backbone from scratch was not feasible within the Colab compute budget. Both pipeline configurations were retained at their default settings as published in the Model Zoo, except for the dataset paths and the number of training steps. Each baseline was trained for 91,800 gradient updates, slightly exceeding the YOLO training budget of 85,800 steps (1,430 training images/batch 5 × 300 epochs) to remain within the same order of magnitude.

We acknowledge that the COCO-pretrained initialisation of SSD and Faster R-CNN gives these baselines an advantage over the from-scratch YOLO models. This makes the YOLO-vs-baseline comparison conservative against YOLO: any precision advantage observed for YOLO is achieved despite this initialisation handicap, and would likely widen if YOLO were also COCO-pretrained.

### Image preprocessing methods

Twelve image preprocessing methods were evaluated for their ability to mitigate the performance degradation caused by overexposure and underexposure: two traditional methods and ten deep-learning-based methods.

#### Traditional methods

- Histogram Equalisation — applied with default OpenCV `cv2.equalizeHist` parameters.
- CLAHE (Contrast Limited Adaptive Histogram Equalisation) — applied with OpenCV defaults: clip limit 2.0, tile grid size 8 × 8. The pseudocode for the full image preprocessing pipeline used to generate the lighting-augmented test sets and the corresponding enhancement outputs is provided in Supplementary Table S1.

#### Deep-learning methods

The ten deep-learning methods listed below were applied using the authors publicly released pretrained weights, with no retraining or fine-tuning on our dataset. This choice ensures that comparisons reflect each method’s intended generalisation behaviour as reported by the original authors. Method names refer to the corresponding cited references in the bibliography:

- Afifi et al.^43^— Multi-scale exposure correction.
- Cui et al.^158^— Lightweight transformer for exposure correction.
- H. Nguyen et al.^152^— Progressive self-enhancement (PSENet).
- H. Wang et al.^151^— Local colour distribution prior (LCDPNet).
- Nsampi et al.^161^— Exposure correction via consistency modelling.
- T. Wang et al.^162^— Ultra-high-definition low-light enhancement.
- Bai et al.^159^— RetinexMamba.
- C. Guo et al.^160^— Zero-DCE.
- Zamir et al.^153^(MIRNet-v2, Contrast Enhancement variant).
- Zamir et al.^153^(MIRNet-v2, Lowlight Enhancement variant).

Each method was applied as a standalone image-to-image transform on each test image before inference. The complete experimental workflow — covering data acquisition, annotation, dataset partitioning, model training, preprocessing, and performance evaluation — is illustrated in Supplementary Figure S1.

### Test set augmentation under simulated lighting conditions

**Fig. 3:**
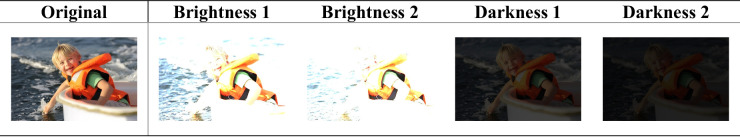
Visual representation of lighting variations in the test dataset. The source image is adapted from the “Life Jacket” dataset by CONTIL, available from Roboflow Universe at https://universe.roboflow.com/contil-rn9fi/life-jacket-kt521 and licensed under CC BY 4.0 (https://creativecommons.org/licenses/by/4.0/). The image was cropped, resized, and enhanced for presentation quality.

To evaluate detector robustness to illumination variation — a common challenge in real-world maritime SAR — the original 100-image test set was augmented into four additional variants by deterministic pixel-intensity adjustments: two simulating overexposure (Brightness 1, Brightness 2) and two simulating underexposure (Darkness 1, Darkness 2). Combined with the unaltered original variant, this yields five lighting conditions for evaluation. Visual samples of the five conditions are shown in Fig. 3. We acknowledge as a limitation that these synthetically perturbed images do not fully capture the complexity of true overexposure or low-light photography (which involve sensor noise characteristics, glare, motion blur, and white-balance shifts that are not modelled by simple intensity scaling); the limitations of synthetic illumination perturbation for generalising to real maritime conditions are discussed in the section “Result discussion”. Pseudocode for the brightness adjustment routine is provided in Supplementary Table S1, and the experimental workflow for evaluating detection models under varying lighting conditions using image enhancement techniques is illustrated in Supplementary Figure S2.

### Evaluation protocol

Evaluation reports four standard object detection metrics: precision, recall, mAP@0.5 (mean Average Precision at IoU threshold 0.5), and mAP@0.5:0.95 (mean Average Precision averaged over IoU thresholds 0.5 to 0.95 in steps of 0.05). For YOLO models, evaluation was performed using the Ultralytics `yolo val` command with default thresholds (confidence 0.001, NMS IoU 0.7, evaluated at the same 320 × 320 resolution as training). For SSD and Faster R-CNN, evaluation was performed using the TensorFlow Object Detection API’s evaluation pipeline with the same IoU thresholds.

Inference speed (frames per second, FPS) was measured separately on the same Tesla T4 GPU with a single-image batch and reported alongside accuracy metrics in the precision–FPS trade-off analysis.

#### Three evaluation slices

Throughout the Results section we report metrics from three distinct evaluation slices, each addressing a different question:

- Validation set (*n* = 100 images, original lighting).

1. Used during training for monitoring and model selection; reported in Table 3.
2. Test set, full (*n* = 100 images, five lighting conditions × twelve preprocessing variants). Used for the main comparative analysis; reported in Tables 4, 5, 6, 7, 8, 9 and 10.
3. Test set, stratified subsamples (5 images per class × 5 random repetitions). Used as a stability check for the precision estimate; reported in Fig. 7.
4. The five subsample means agree with the full-test-set values reported in Table 4 (Original lighting condition) within the observed run-to-run variability.

**Table 3:** Overall Detection Accuracy and Inference Efficiency of YOLO Versions, SSD, and Faster R-CNN on the Custom Life Jacket Dataset.

All	Precision	Recall	mAP50	mAP50-95
YOLOv5	0.535	0.572	0.553	0.372
YOLOv6	0.522	0.603	0.577	0.399
YOLOv7	0.567	0.601	0.577	0.373
YOLOv8	0.568	0.514	0.596	0.413
YOLOv9	0.633	0.492	0.591	0.425
YOLOv10	0.63	0.499	0.565	0.401
YOLOv11	0.571	0.58	0.595	0.425
YOLOv12	0.569	0.424	0.525	0.356
SSD	0.449	0.594	0.485	0.273
Faster R-CNN	0.481	0.634	0.508	0.357

**Table 4:** Precision under five lighting conditions (Original, Brightness 1/2, Darkness 1/2) without preprocessing for all evaluated detectors.

	Model	Precision	Recall	mAP50	mAP50-95
Original	YOLOv5	0.734	0.661	0.753	0.517
	YOLOv6	0.647	0.696	0.717	0.495
	YOLOv7	0.747	0.708	0.761	0.476
	YOLOv8	0.754	0.679	0.765	0.516
	YOLOv9	0.64	0.671	0.719	0.518
	YOLOv10	0.678	0.73	0.769	0.557
	YOLOv11	0.794	0.668	0.767	0.526
	YOLOv12	0.803	0.618	0.752	0.532
	SSD	0.673	0.658	0.706	0.441
	Faster R-CNN	0.639	0.688	0.654	0.452
Brightness 1	YOLOv5	0.454	0.406	0.409	0.208
	YOLOv6	0.517	0.378	0.404	0.223
	YOLOv7	0.36	0.44	0.356	0.171
	YOLOv8	0.45	0.452	0.434	0.226
	YOLOv9	0.507	0.329	0.326	0.158
	YOLOv10	0.466	0.334	0.413	0.218
	YOLOv11	0.54	0.341	0.397	0.212
	YOLOv12	0.418	0.414	0.394	0.206
	SSD	0.397	0.519	0.449	0.238
	Faster R-CNN	0.378	0.481	0.432	0.246
Brightness 2	YOLOv5	0.34	0.284	0.244	0.11
	YOLOv6	0.371	0.286	0.25	0.125
	YOLOv7	0.288	0.211	0.143	0.0666
	YOLOv8	0.341	0.258	0.229	0.11
	YOLOv9	0.311	0.178	0.155	0.0648
	YOLOv10	0.375	0.213	0.248	0.113
	YOLOv11	0.26	0.249	0.191	0.0892
	YOLOv12	0.294	0.279	0.239	0.118
	SSD	0.242	0.429	0.334	0.153
	Faster R-CNN	0.176	0.316	0.201	0.108
Darkness 1	YOLOv5	0.572	0.623	0.585	0.376
	YOLOv6	0.52	0.527	0.546	0.336
	YOLOv7	0.669	0.578	0.656	0.395
	YOLOv8	0.621	0.589	0.623	0.39
	YOLOv9	0.613	0.651	0.651	0.429
	YOLOv10	0.662	0.566	0.652	0.457
	YOLOv11	0.577	0.649	0.642	0.419
	YOLOv12	0.607	0.558	0.581	0.372
	SSD	0.201	0.445	0.244	0.115
	Faster R-CNN	0.471	0.566	0.488	0.331
Darkness 2	YOLOv5	0.24	0.244	0.211	0.14
	YOLOv6	0.207	0.117	0.143	0.0929
	YOLOv7	0.58	0.415	0.4	0.237
	YOLOv8	0.222	0.287	0.224	0.136
	YOLOv9	0.38	0.285	0.273	0.163
	YOLOv10	0.198	0.312	0.232	0.136
	YOLOv11	0.506	0.143	0.184	0.0989
	YOLOv12	0.171	0.168	0.145	0.0855
	SSD	0.006	0.190	0.014	0.004
	Faster R-CNN	0.149	0.241	0.154	0.099

**Table 5:** Comparative Model Accuracy Under Normal Conditions: Impact of Preprocessing Methods.

Original Condition		YOLOv5	YOLOv6	YOLOv7	YOLOv8	YOLOv9	YOLOv10	YOLOv11	YOLOv12	SSD	Faster *R*-CNN
Default		0.734	0.647	0.747	0.754	0.64	0.678	**0.794**	0.803	0.673	0.639
Histogram		0.743	0.604	0.755	0.727	0.689	0.727	0.793	**0.811**	0.672	0.642
Clahe		0.591	0.681	0.7	0.648	0.72	0.745	0.709	0.808	0.608	0.621
Nsampi et al^161^		0.748	0.657	0.729	0.676	0.588	0.75	0.689	0.788	0.627	0.633
Cui et al.^158^		0.73	0.673	0.7	0.705	0.59	**0.786**	0.734	0.674	0.591	0.586
H. Nguyen et al.^152^		**0.787**	0.582	**0.778**	0.732	0.619	0.748	0.765	0.657	0.657	**0.649**
H. Wang et al.^151^		0.757	0.595	0.705	0.701	0.668	0.656	0.763	**0.811**	0.632	0.607
Afifi et al.^43^		0.712	0.571	0.701	**0.805**	**0.74**	0.731	0.685	0.76	0.601	0.636
T. Wang et al.^162^		0.719	0.614	0.719	0.685	0.665	0.766	0.775	0.687	0.646	0.625
Bai et al.^159^		0.683	0.626	0.621	0.734	0.712	0.617	0.674	0.69	0.565	0.605
C. Guo et al.^160^		0.637	**0.692**	0.755	0.734	0.69	0.743	0.737	0.737	**0.674**	0.614
Zamir et al.^153^	Contrast Enhancement	0.674	0.617	0.607	0.732	0.65	0.704	0.747	0.776	0.612	0.585
	Lowlight Enhancement	0.725	0.54	0.577	0.683	0.569	0.724	0.741	0.647	0.562	0.542

Bold values indicate the highest precision in each column.

**Table 6:** Model Accuracy Under Brightness1 Conditions Across Preprocessing Techniques and Detection Architectures.

Brightness1 Condition		YOLOv5	YOLOv6	YOLOv7	YOLOv8	YOLOv9	YOLOv10	YOLOv11	YOLOv12	SSD	Faster *R*-CNN
Default		0.454	0.517	0.36	0.45	0.507	0.466	0.54	0.418	0.397	0.378
Histogram		0.461	**0.533**	0.426	0.495	0.508	0.483	0.534	0.441	0.362	0.364
Clahe		0.434	0.431	0.385	**0.549**	0.534	0.531	0.527	0.39	0.381	0.389
Nsampi et al.^161^		0.521	0.501	0.418	0.537	0.526	0.646	0.628	0.491	0.401	0.466
Cui et al.^158^		0.369	0.453	0.358	0.433	0.478	0.609	0.48	**0.517**	0.416	0.419
H. Nguyen et al.^152^		0.483	0.476	0.428	0.476	0.54	**0.652**	0.542	0.463	0.347	0.357
H. Wang et al.^151^		0.473	0.433	0.354	0.469	0.481	0.551	**0.631**	0.469	0.373	0.45
Afifi et al.^43^		**0.566**	0.508	**0.569**	0.537	**0.537**	0.586	**0.631**	0.507	**0.419**	**0.496**
T. Wang et al.^162^		0.446	0.495	0.338	0.483	0.454	0.6	0.502	0.431	0.288	0.33
Bai et al.^159^		0.402	0.318	0.378	0.172	0.149	0.285	0.166	0.318	0.207	0.197
C. Guo et al.^160^		0.434	0.343	0.3	0.409	0.449	0.527	0.502	0.431	0.323	0.285
Zamir et al.^153^	Contrast Enhancement	0.446	0.357	0.41	0.38	0.375	0.335	0.33	0.37	0.318	0.233
	Lowlight Enhancement	0.301	0.402	0.155	0.407	0.301	0.135	0.233	0.274	0.25	0.114

Bold values indicate the highest precision in each column.

**Table 7:** Comparative Model Accuracy Under Brightness2 Conditions: Impact of Preprocessing Methods.

Brightness2 Condition		YOLOv5	YOLOv6	YOLOv7	YOLOv8	YOLOv9	YOLOv10	YOLOv11	YOLOv12	SSD	Faster *R*-CNN
Default		0.34	0.371	0.288	0.341	0.311	0.375	0.26	0.294	0.242	0.176
Histogram		0.307	0.353	0.208	0.324	0.305	0.389	0.229	0.275	0.231	0.153
Clahe		0.335	0.414	0.328	0.293	0.234	0.341	0.268	0.29	0.298	0.216
Nsampi et al.^161^		0.381	**0.517**	0.393	0.318	0.325	0.449	**0.46**	0.347	0.316	0.222
Cui et al.^158^		0.349	0.306	0.255	0.304	0.269	0.375	0.33	0.268	0.257	0.207
H. Nguyen et al.^152^		**0.395**	0.472	0.287	0.274	0.294	0.426	0.367	0.237	0.248	0.214
H. Wang et al.^151^		0.35	0.358	0.291	0.348	0.33	0.515	0.373	0.343	0.251	0.242
Afifi et al.^43^		0.393	0.486	**0.498**	**0.51**	**0.416**	**0.57**	0.416	**0.469**	**0.317**	**0.32**
T. Wang et al.^162^		0.382	0.397	0.307	0.473	0.385	0.203	0.31	0.343	0.203	0.131
Bai et al.^159^		0.349	0.0561	0.107	0.152	0.0709	0.167	0.0809	0.082	0.098	0.057
C. Guo et al.^160^		0.314	0.334	0.355	0.388	0.346	0.422	0.327	0.31	0.205	0.122
Zamir et al.^153^	Contrast Enhancement	0.255	0.401	0.177	0.342	0.345	0.421	0.272	0.216	0.177	0.106
	Lowlight Enhancement	0.25	0.28	0.083	0.113	0.0413	0.0777	0.19	0.252	0.117	0.047

Bold values indicate the highest precision in each column.

**Table 8:** Comparative Model Accuracy Under Darkness1 Conditions: Impact of Preprocessing Methods.

Darkness1 Condition		YOLOv5	YOLOv6	YOLOv7	YOLOv8	YOLOv9	YOLOv10	YOLOv11	YOLOv12	SSD	Faster *R*-CNN
Default		0.572	0.52	0.669	0.621	0.613	0.662	0.577	0.607	0.201	0.471
Histogram		0.697	0.639	0.781	0.735	0.618	0.729	0.788	**0.787**	0.64	**0.651**
Clahe		**0.775**	0.597	0.748	0.677	0.657	0.744	**0.812**	0.758	0.493	0.644
Nsampi et al.^161^		0.68	0.67	0.71	0.67	0.608	0.678	0.715	0.711	0.568	0.625
Cui et al.^158^		0.612	**0.739**	0.668	0.683	0.619	0.661	0.69	0.673	0.486	0.575
H. Nguyen et al.^152^		0.656	0.596	0.76	0.712	0.649	0.65	0.687	0.716	0.549	0.615
H. Wang et al.^151^		0.699	0.628	0.678	0.667	**0.737**	0.661	0.75	0.741	0.542	0.637
Afifi et al.^43^		0.64	0.709	0.604	0.724	0.592	**0.783**	0.671	0.682	0.588	0.582
T. Wang et al.^162^		0.678	0.642	0.73	0.683	0.684	0.746	0.759	0.705	**0.644**	0.638
Bai et al.^159^		0.717	0.597	0.711	**0.761**	0.604	0.755	0.733	0.675	0.606	0.64
C. Guo et al.^160^		0.605	0.574	0.759	0.688	0.658	0.718	0.685	0.757	0.536	0.61
Zamir et al.^153^	Contrast Enhancement	0.752	0.616	0.688	0.745	0.711	0.699	0.725	0.721	0.596	0.644
	Lowlight Enhancement	0.712	0.695	**0.81**	0.701	0.634	0.747	0.748	0.703	0.619	0.634

Bold values indicate the highest precision in each column.

**Table 9:** Comparative Model Accuracy Under Darkness2 Conditions: Impact of Preprocessing Methods.

Darkness2 Condition		YOLOv5	YOLOv6	YOLOv7	YOLOv8	YOLOv9	YOLOv10	YOLOv11	YOLOv12	SSD	Faster *R*-CNN
Default		0.24	0.207	0.58	0.222	0.38	0.198	0.506	0.171	0.006	0.149
Histogram		**0.726**	0.634	0.675	0.665	0.648	0.704	0.733	0.673	**0.619**	0.647
Clahe		0.64	0.612	0.58	0.633	0.695	0.712	0.66	0.665	0.493	0.542
Nsampi et al.^161^		0.623	**0.739**	0.701	0.616	0.594	0.717	0.608	0.688	0.493	0.595
Cui et al.^158^		0.647	0.59	0.657	0.639	0.7	0.563	0.658	0.597	0.364	0.449
H. Nguyen et al.^152^		0.667	0.693	0.629	0.727	0.618	0.756	0.67	0.659	0.495	0.625
H. Wang et al.^151^		0.677	0.673	0.63	0.601	0.679	0.799	0.71	0.659	0.475	0.613
Afifi et al.^43^		0.68	0.655	0.683	0.674	0.558	0.675	0.627	0.684	0.549	0.633
T. Wang et al.^162^		0.611	0.54	0.687	0.67	0.723	0.745	0.714	0.676	0.54	0.64
Bai et al.^159^		0.65	0.663	0.55	0.636	0.715	**0.804**	0.692	0.633	0.524	**0.66**
C. Guo et al.^160^		0.703	0.623	0.658	0.699	**0.741**	0.789	0.706	0.673	0.477	0.605
Zamir et al.^153^	Contrast Enhancement	0.665	0.611	**0.738**	0.611	0.729	0.641	**0.745**	0.621	0.472	0.601
	Lowlight Enhancement	0.684	0.593	0.732	**0.735**	0.622	0.763	0.688	**0.734**	0.54	0.637

Bold values indicate the highest precision in each column.

**Table 10:** Complete Preprocessing Analysis for YOLOv10 (precision from baseline).

Method		Normal	Brightness1	Brightness2	Darkness1	Darkness2
Default		0.678	0.466	0.375	0.662	0.198
Histogram		7.23%	3.65%	3.73%	10.12%	255.56%
CLAHE		9.88%	13.95%	−9.07%	12.39%	259.60%
Nsampi et al.^161^		10.62%	38.63%	19.73%	2.42%	262.12%
Cui et al.^158^		15.93%	30.69%	0.00%	−0.15%	184.34%
H. Nguyen et al.^152^		10.32%	39.91%	13.60%	−1.81%	281.82%
H. Wang et al.^151^		−3.24%	18.24%	37.33%	−0.15%	303.54%
Afifi et al.^43^		7.82%	25.75%	52.00%	18.28%	240.91%
T. Wang et al.^162^		12.98%	28.76%	−45.87%	12.69%	276.26%
Bai et al.^159^		−8.97%	−38.84%	−55.47%	14.05%	306.06%
C. Guo et al.^160^		9.59%	13.09%	12.53%	8.46%	298.49%
Zamir et al.^153^	Contrast Enhancement	3.83%	−28.11%	12.27%	5.59%	223.74%
	Lowlight Enhancement	6.78%	−71.03%	−79.28%	12.84%	285.35%

Readers comparing values between Table 3; Fig. 7, and Table 4 should note that small numerical differences arise from these distinct evaluation slices rather than from inconsistent results.

#### Statistical analysis

Because compute constraints precluded multi-seed retraining of all ten detectors across all conditions, we instead characterise uncertainty through bootstrap resampling of the test set predictions. For each model × lighting × preprocessing combination, the per-image prediction outputs were saved (`yolo val --save-json` for YOLO, equivalent JSON export for the TF-OD baselines). 95% bootstrap confidence intervals were computed for the threshold-independent metrics mAP@0.5 and mAP@0.5:0.95 by resampling the 100 test images with replacement 1,000 times and recomputing each metric on each resample; CIs are reported alongside point estimates in the headline summary table (Table 11). Precision and recall are reported as point estimates at the F1-optimal confidence threshold per class (Ultralytics default) to retain operational interpretability; bootstrap CIs are not reported for these threshold-dependent metrics without further specification of the deployment operating point. For headline pairwise comparisons (e.g., YOLOv10 vs. other YOLO versions; preprocessed vs. non-preprocessed performance under a given lighting condition), we additionally report Wilcoxon signed-rank statistics across the four life jacket classes × five lighting conditions = 20 paired observations. We acknowledge that single-run training with bootstrap test-set confidence intervals is a weaker form of uncertainty quantification than multi-seed retraining and is identified as a limitation in the section “Result discussion”.

**Table 11:** Headline detection performance for YOLOv10 across the five lighting conditions, comparing no-preprocessing baseline against the best-performing preprocessing method (selected by mAP@0.5). Values show point estimate with 95% bootstrap confidence interval (1,000 resamples of the 100 test images).

Lighting condition	Preprocessing method	mAP@0.5 [95% CI]	mAP@0.5:0.95 [95% CI]
Original	(none)	0.752 [0.667, 0.858]	0.534 [0.469, 0.620]
Original	Histogram Equalisation	0.746 [0.663, 0.853]	0.529 [0.461, 0.617]
Brightness 1	(none)	0.377 [0.307, 0.457]	0.191 [0.155, 0.245]
Brightness 1	Afifi et al.	0.448 [0.380, 0.539]	0.257 [0.210, 0.319]
Brightness 2	(none)	0.209 [0.155, 0.289]	0.090 [0.060, 0.139]
Brightness 2	Afifi et al.	0.286 [0.225, 0.377]	0.164 [0.128, 0.212]
Darkness 1	(none)	0.637 [0.559, 0.756]	0.437 [0.373, 0.532]
Darkness 1	Histogram Equalisation	0.753 [0.675, 0.859]	0.523 [0.462, 0.614]
Darkness 2	(none)	0.219 [0.161, 0.308]	0.128 [0.096, 0.186]
Darkness 2	Bai et al. (RetinexMamba)	0.709 [0.636, 0.815]	0.475 [0.413, 0.569]

### Use of generative AI tools

During the preparation of this work, the authors used ChatGPT (OpenAI) and Claude (Anthropic) to improve readability and language. These tools were not used to generate, analyse, or interpret experimental data, nor to draw scientific conclusions. After using these tools, the authors reviewed and edited the content as needed and take full responsibility for the content of the publication.

## Experiment result

To systematically evaluate the effectiveness of our object detection framework, this section presents a series of quantitative and qualitative experiments using the YOLO family of models, SSD, and Faster R-CNN. The evaluation spans multiple dimensions, including model performance across object categories, robustness under diverse lighting conditions, and the influence of various preprocessing techniques. Visual and numerical comparisons highlight model behaviors in scenarios ranging from well-lit environments to extreme overexposure and underexposure.

To systematically evaluate the YOLO model’s performance, we implemented a standardized evaluation workflow based on commonly accepted object detection metrics. This process involves defining key evaluation parameters (e.g., task type, data split, image resolution, confidence threshold, and IoU), loading the trained model and dataset, and executing the evaluation routine using the Ultralytics framework. The evaluation outputs include precision, recall, mAP at IoU 0.5 (mAP@0.5), and mAP averaged over IoU thresholds from 0.5 to 0.95 (mAP@0.5:0.95), which together offer a comprehensive assessment of detection accuracy and localization quality. Supplementary Table S2 outlines the complete evaluation workflow used in this experiment, from setup to result extraction, ensuring repeatability and consistency across model comparisons.

To complement the evaluation phase, we implemented a standardized inference pipeline to test the trained YOLO model on unseen images. This procedure involves loading the trained weights, passing input images through the model, and extracting detection outputs such as bounding boxes, confidence scores, and class predictions. Post-processing steps were applied to filter low-confidence detections based on a predefined threshold and to visualize the results. Supplementary Table S3 summarizes the complete inference workflow, detailing each step from model initialization to result visualization and storage, thereby ensuring transparency and reproducibility in the detection process.

**Fig. 4:**
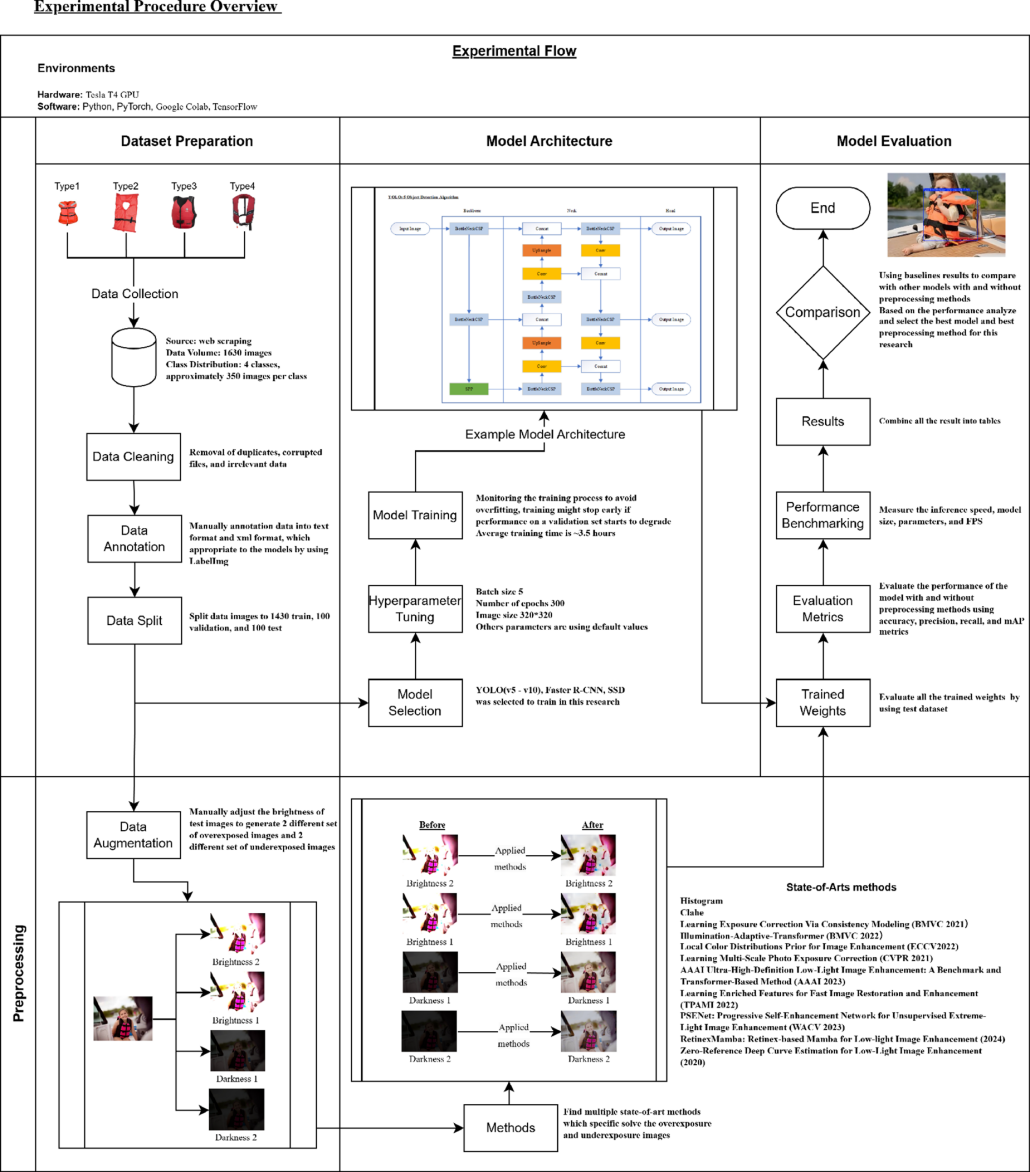
Overview of the experimental procedure. The source images are adapted from the “Life Jacket” dataset by CONTIL, available from Roboflow Universe at https://universe.roboflow.com/contil-rn9fi/life-jacket-kt521 and licensed under CC BY 4.0 (https://creativecommons.org/licenses/by/4.0/). Images were cropped, resized, and enhanced for presentation quality.

To establish a clear and reproducible methodology, Fig. 4 presents a comprehensive overview of the experimental procedure. The workflow begins with dataset preparation, which includes data collection from diverse sources, manual annotation, and class-balanced splitting. The selected models—YOLOv5 to YOLOv12, SSD, and Faster R-CNN—were trained using standardized hyperparameters and monitored to avoid overfitting. A key component of this pipeline is the application of state-of-the-art image preprocessing techniques to simulate overexposed and underexposed conditions, enhancing model robustness in real-world scenarios. The evaluation phase then benchmarks each model’s performance using precision, recall, mAP, and FPS across both original and enhanced datasets. This figure serves as a visual roadmap for the experimental design discussed in the subsequent sections.

The experimental results presented across this section provide a comprehensive evaluation of model performance under standard and challenging visual conditions. From baseline comparisons on clean datasets to rigorous assessments under lighting distortions, the findings highlight key trade-offs between detection accuracy and inference efficiency across different architectures. Notably, the integration of preprocessing techniques demonstrated tangible improvements in model robustness, particularly in low-visibility scenarios. These outcomes affirm the importance of combining strong baseline models with targeted image enhancement strategies to ensure reliable detection performance in real-world applications, especially in safety-critical environments like maritime operations.

### Comparison of YOLO versions & SSD & faster R-CNN in custom dataset (life jacket)

Throughout the following analyses, we report results from three distinct evaluation slices to address different questions. First, Table 3 reports end-of-training validation-set performance (*n* = 100 validation images) used during model development. Second, Fig. 7 reports a precision–FPS trade-off based on stratified random subsamples of the test set (5 images per class × 5 repetitions); these subsamples were used as a stability check during preliminary analysis and the resulting precision values are consistent with the full-test-set evaluation reported in Table 4 within the observed run-to-run variability. Third, Table 4 and the subsequent Tables 5, 6, 7, 8 and 9, report full test-set performance (*n* = 100 test images) under five lighting conditions and across preprocessing methods. Readers comparing values between Table 3; Fig. 7, and Table 4 should note that small numerical differences arise from these distinct evaluation slices rather than inconsistent results. To establish a baseline comparison of the overall performance of the evaluated object detection models, we first examined their detection accuracy on the full custom life jacket dataset. This evaluation considers key metrics including precision, recall, mAP@0.5, and mAP@0.5:0.95 to capture both localization and classification performance. As shown in Table 3, YOLOv9 and YOLOv10 achieved the highest precision (0.633 and 0.630, respectively), while YOLOv8 obtained the highest mAP@0.5:0.95 (0.413), indicating strong detection consistency across IoU thresholds. Although Faster R-CNN exhibited the highest recall (0.634), its overall mAP and precision were lower than those of the latest YOLO models. SSD, while slightly faster in inference time (as shown in Fig. 7), had the lowest mAP@0.5:0.95 (0.273), making it less suitable for high-accuracy detection tasks. These findings highlight the superior accuracy of modern YOLO versions, particularly YOLOv9 and YOLOv10, when applied to safety-critical detection scenarios.

**Fig. 5:**
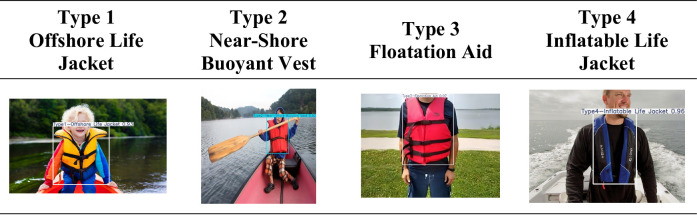
Visual detection results for different Types of four life jacket categories: Type 1 (Offshore Life Jacket), Type 2 (Near-Shore Buoyant Vest), Type 3 (Floatation Aid), and Type 4 (Inflatable Life Jacket). Sample images from “Life Jacket” by CONTIL, available at https://universe.roboflow.com/contil-rn9fi/life-jacket-kt521 under CC BY 4.0 (https://creativecommons.org/licenses/by/4.0/). Images were cropped, resized, and enhanced for quality.

Visual detection performance often varies across object categories, especially in safety-critical applications like life jacket identification. To understand how different life jacket types appear in detection scenarios, we curated a custom dataset with representative samples of four distinct categories: Type 1 (Offshore Life Jacket), Type 2 (Near-Shore Buoyant Vest), Type 3 (Floatation Aid), and Type 4 (Inflatable Life Jacket). Figure 5 presents example detection outputs for each category using the trained object detection model. These visual samples help illustrate the appearance and structural variation among life jacket types, providing context for the subsequent performance analysis.

**Fig. 6:**
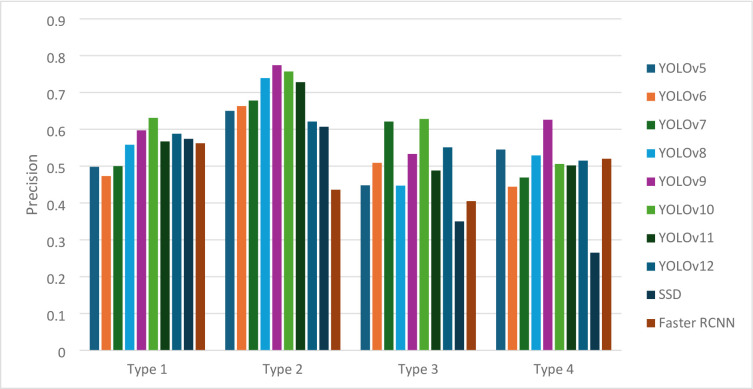
Precision Scores for Different Types of Life Jackets Across YOLO Versions, SSD, and Faster R-CNN.

To gain deeper insight into how each object detection model performs across different types of life jackets, a detailed per-class evaluation of detection precision was conducted. This analysis is critical for safety-critical applications where consistent recognition across all equipment types is essential. As illustrated in Fig. 6, YOLOv10 ranks among the top detectors across all four life jacket categories, with the strongest aggregate performance in visually challenging scenarios. YOLOv9 also performs reliably, particularly for Type 2 and Type 3, indicating its stability across mid-complexity classes. In contrast, SSD and Faster R-CNN show more variability in class-wise performance—SSD exhibits notably low precision for Type 4, while Faster R-CNN, despite delivering high recall in some cases, suffers from lower precision and overall accuracy. These results underscore the superiority of recent YOLO versions in handling intra-class variability and reinforce their suitability for real-world deployment where robust, category-consistent detection is critical.

To contextualize our contributions, Table 12 presents a comparative analysis with existing life jacket detection research. While direct quantitative comparison is challenging due to varying datasets and evaluation protocols, we provide qualitative assessment across key dimensions:

**Table 12:** Comparative analysis with existing similar life jacket detection research.

Study	Dataset Size	Model	Lighting Focus	mAP	Platform	Multi-class	Preprocessing
^66^	812 images	Custom CNN	Normal only	76.30%	UAV	No (binary)	None
^67^	Synthetic	YOLOv4	Daylight	68.50%	UAV	No	None
^21^	54,000 frames	YOLOv5	Various	71.20%	UAV	Yes (5 classes)	None
^95^	1,200 images	U-Net	Indoor/Outdoor	93%	Fixed camera	No	None
^17^	8,000 images	YoloOW	Daylight	73.80%	UAV	Yes (2 classes)	None
Our work	1,630 images	YOLOv5-12, SSD, Faster R-CNN	5 conditions	76.90%	Multiple	Yes (4 types)	13 methods

Our work advances the field in several key aspects:

- We provide the first systematic evaluation of multiple YOLO versions (v5-v10) for life jacket detection, identifying YOLOv10 as optimal for this application.
- Unlike previous studies focusing solely on daylight conditions, we explicitly address extreme lighting through comprehensive preprocessing evaluation, achieving up to 60% improvement in darkness conditions.
- We distinguish between four life jacket types rather than treating them as a single class, providing granular performance analysis crucial for equipment-specific SAR protocols;
- Our multi-platform approach (suitable for vessels, drones, and fixed cameras) offers broader applicability than UAV-specific solutions.

While previous works have made valuable contributions, particularly in UAV-based detection and synthetic data generation, they have not addressed the critical challenge of lighting robustness that we tackle through systematic preprocessing integration^21^. SeaDronesSee dataset remains the largest, but focuses on general maritime objects rather than detailed life jacket categorization. Ye and Li^95^ achieve high accuracy using semantic segmentation but require significantly more computational resources than our real-time YOLO approach. Our work thus fills a crucial gap by providing a lightweight, multi-condition, multi-class detection system with demonstrated robustness across challenging maritime environments.

**Fig. 7:**
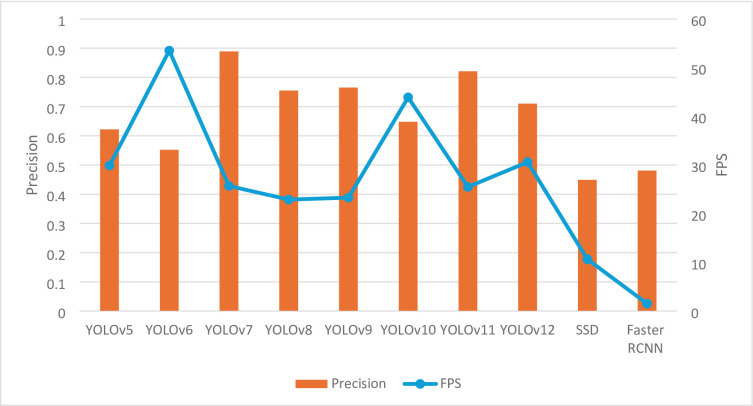
Precision versus inference speed (FPS) trade-off for all evaluated detectors on the original-lighting test set. Higher and rightmost is better.

A comprehensive evaluation of each model’s deployment feasibility requires analysing both detection accuracy and computational efficiency. As shown in Fig. 7, the relationship between precision and frames per second (FPS) offers a clear view of how each architecture balances performance and real-time capability. YOLOv10 emerges as the optimal choice, achieving an exceptional balance with strong precision 0.648 while maintaining competitive inference speed (43.9 FPS). This combination makes it particularly suitable for latency-sensitive applications such as maritime safety and rescue operations where both accuracy and real-time processing are critical. While YOLOv6 demonstrates the highest FPS (53.5 FPS), its precision drops to 0.552, illustrating the typical trade-off between speed and accuracy. YOLOv7 offers the highest precision 0.889 but at a reduced speed (25.7 FPS), whereas YOLOv11 and other YOLO variants maintain relatively consistent precision levels 0.755–0.821 with moderate speeds (22.9–25.5 FPS). In contrast, Faster R-CNN, despite reasonable precision 0.481, suffers from extremely low FPS (1.52 FPS), due to its high model complexity and inference latency, rendering it impractical for real-time tasks. SSD performs marginally better than Faster R-CNN in terms of speed (10.65 FPS) but with lower precision 0.449, trailing behind all YOLO variants in both metrics. These results reinforce YOLOv10 position as the most balanced architecture, offering robust detection performance without significantly compromising real-time responsiveness, a critical requirement for maritime safety applications.

In summary, the evaluation on the custom life jacket dataset reveals distinct performance characteristics across the tested object detection models. While two-stage detectors like Faster R-CNN demonstrated reasonable detection capability, their severe inference latency (1.52 FPS) makes them entirely unsuitable for real-time deployment. Lightweight models such as SSD offered improved processing speeds but remained inadequate for real-time applications while also sacrificing detection precision. The YOLO family consistently outperformed other architectures, with YOLOv10 emerging as the optimal solution by achieving the best balance between high precision 0.648 and real-time processing capability (43.9 FPS). While YOLOv7 achieved the highest precision 0.889, and YOLOv6 delivered faster inference (53.5 FPS), YOLOv10 balanced performance profile makes it uniquely suited for maritime safety applications where both detection reliability and responsive operation are non-negotiable requirements. This superior trade-off between accuracy and speed, combined with its consistent performance across varying life jacket types, establishes YOLOv10 as the most practical choice for real-world deployment. The next section further investigates how these models, particularly YOLOv10, perform under challenging visual conditions.

### Comparison of YOLO versions & SSD & faster R-CNN under different lighting conditions

To build upon the previous evaluation conducted under normal lighting conditions, this section explores how object detection performance varies when exposed to challenging illumination scenarios, such as overexposed and underexposed environments. Real-world maritime search and rescue missions often face highly dynamic lighting, which can severely impact model accuracy. Therefore, to assess the robustness of each detection model, a series of controlled experiments were conducted using five different lighting conditions: original, brightness1, brightness2, darkness1, and darkness2. Each model (YOLOv5 through YOLOv12, SSD, and Faster R-CNN) was benchmarked on this augmented test dataset to evaluate its ability to maintain detection reliability across fluctuating visibility levels. The analysis that follows highlights the inherent limitations of some models and reveals which architectures demonstrate greater resilience under non-ideal lighting, offering critical insights into model suitability for deployment in real-world maritime environments.

**Fig. 8:**
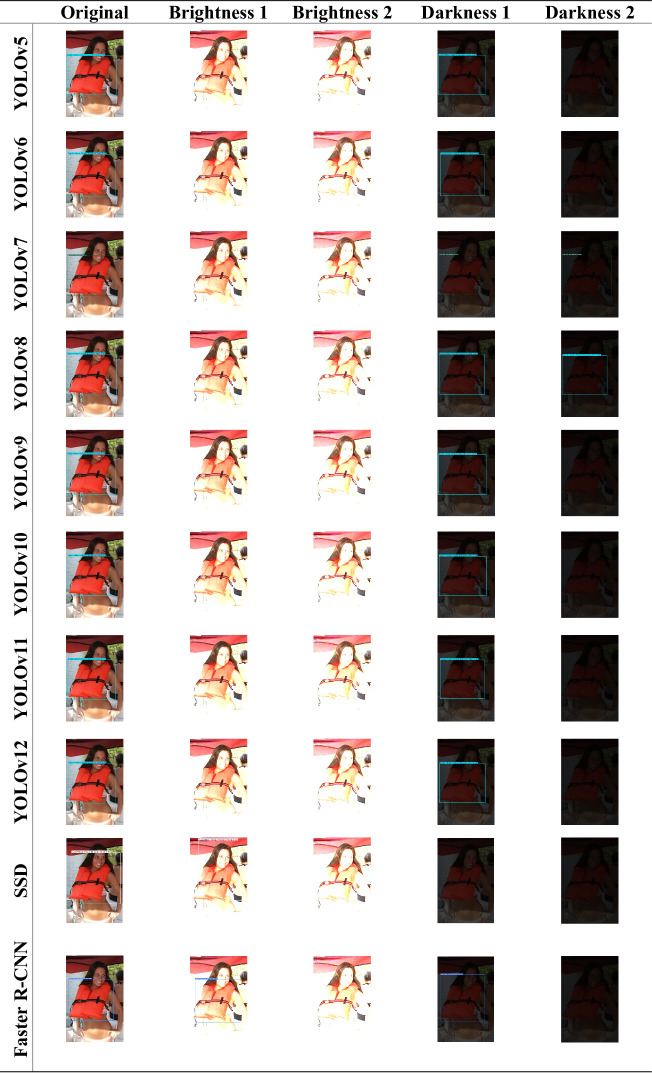
Detection outputs from YOLOv5–YOLOv12, SSD, and Faster R-CNN across five lighting scenarios (Original, Brightness 1/2, Darkness 1/2) on a representative test image, without preprocessing. Sample images from “Life Jacket” by CONTIL, available at https://universe.roboflow.com/contil-rn9fi/life-jacket-kt521 under CC BY 4.0 (https://creativecommons.org/licenses/by/4.0/). Images were cropped, resized, and enhanced for quality.

To gain qualitative insights into how lighting variations impact detection reliability, a visual comparison was conducted across all evaluated models without applying any preprocessing enhancements. As shown in Fig. 8, the detection outputs of YOLOv5 through YOLOv12, SSD, and Faster R-CNN were assessed under five lighting scenarios: the original, two levels of overexposure (Brightness 1 and 2), and two levels of underexposure (Darkness 1 and 2). The results reveal a significant degradation in object localization and classification performance as lighting deviates from ideal conditions. Under overexposed settings, YOLOv5 to YOLOv8 and YOLOv11 to YOLOv12 often fail to produce bounding boxes, while SSD and Faster R-CNN typically miss detections altogether, indicating their susceptibility to pixel saturation. YOLOv9 and YOLOv10, however, demonstrate greater robustness, maintaining more consistent predictions despite the extreme exposure. In darker conditions, detection quality continues to deteriorate—SSD and Faster R-CNN provide no predictions, while earlier YOLO versions show partial or inaccurate detections. Notably, YOLOv10 consistently detects the life jacket even under severe underexposure, though with diminished box clarity. These qualitative results highlight the limitations of traditional models in poor lighting environments and underscore the architectural improvements in newer YOLO versions, especially YOLOv10, which shows strong resilience in challenging visibility scenarios.

To support the qualitative findings, a detailed quantitative evaluation was performed across all models under five lighting conditions: original, two overexposures, and two underexposures. As shown in Table 4, detection performance consistently declines as lighting deviates from normal conditions, with the most severe drops observed under Brightness 2 and Darkness 2. YOLOv8 and YOLOv10 demonstrate relatively stable performance, particularly under mild underexposure, indicating stronger resilience to illumination shifts. In contrast, SSD and Faster R-CNN suffer sharp performance degradation, often failing to produce reliable predictions under extreme lighting. These results underscore the importance of model robustness when deploying object detection systems in unpredictable or harsh visual environments.

**Fig. 9:**
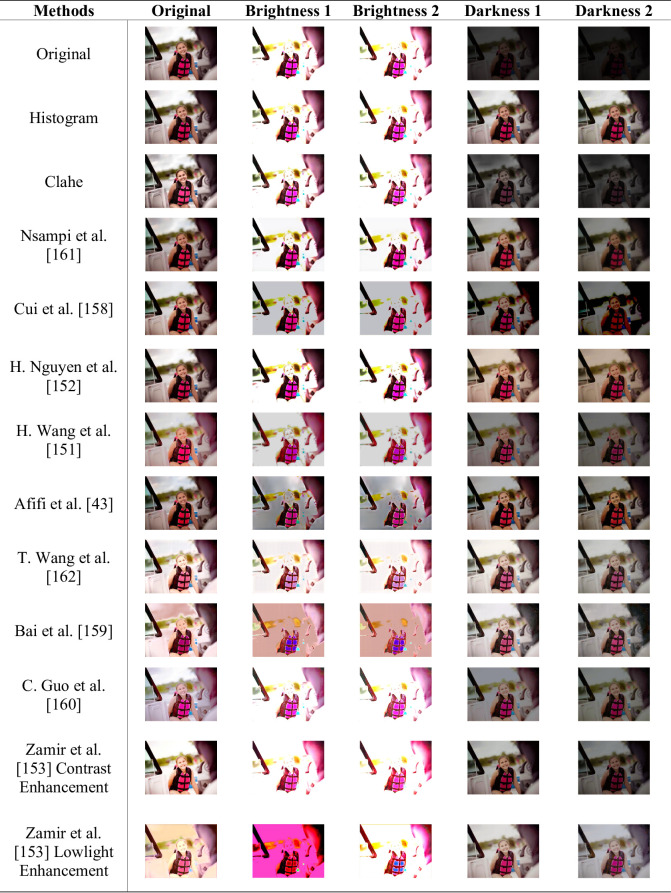
Comparison of lighting adjustment techniques under varying exposure conditions, highlighting their effectiveness in enhancing image clarity and preserving details for object detection. Sample images from “Life Jacket” by CONTIL, available at https://universe.roboflow.com/contil-rn9fi/life-jacket-kt521 under CC BY 4.0 (https://creativecommons.org/licenses/by/4.0/). Images were cropped, resized, and enhanced for quality.

To evaluate the potential of preprocessing techniques in mitigating the adverse effects of lighting distortions, Fig. 9 presents a visual comparison of various state-of-the-art image enhancement methods applied across diverse illumination scenarios. This figure serves to justify the role of preprocessing within the object detection pipeline by demonstrating how these techniques restore visibility and preserve structural features in overexposed and underexposed images. The methods include both traditional techniques like Histogram Equalization and CLAHE, as well as deep learning-based approaches from Afifi et al.^43^, Cui et al.^158^, and Zamir et al.^153^ As illustrated, deep learning-based methods generally provide superior illumination correction with minimal visual artifacts, especially under extreme lighting. In contrast, traditional methods may overcompensate, resulting in unnatural contrast or noise amplification. These observations highlight the importance of selecting preprocessing algorithms that enhance image clarity without compromising visual integrity, ultimately improving downstream object detection performance in challenging environments.

To assess the impact of preprocessing methods under ideal conditions, we evaluated detection accuracy across multiple models using the original (unaltered) test dataset. Table 5, presents a comparative analysis of precision scores achieved by each model when paired with various enhancement techniques. The results reveal that learning-based methods, such as those proposed by Afifi et al.^43^, Cui et al.^158^, and H. Nguyen et al.^152^, consistently improve detection accuracy across most YOLO versions. Notably, YOLOv10 combined with Cui et al.^158^’s method achieves one of the highest overall precision scores, while YOLOv8 paired with Afifi et al.^43^ also shows strong performance. In contrast, traditional approaches like CLAHE and lowlight enhancement by Zamir et al.^153^ exhibit more variable results, sometimes even reducing model accuracy. These findings suggest that certain preprocessing techniques can enhance model robustness even in normal lighting, reinforcing their value beyond adverse conditions.

Detection performance under moderately overexposed conditions reveals notable variations in how each object detection model responds to brightness distortion when paired with different preprocessing techniques. As reported in Table 6, YOLOv10 is among the top-performing detectors under moderately overexposed conditions, achieving the highest precision when combined with techniques from Nsampi et al.^161^, Cui et al.^158^, and H. Nguyen et al.^152^ These combinations outperform the default configuration and showcase robust adaptability to lighting stress. Afifi et al.^43^’s method also contributes to accuracy improvements across multiple YOLO versions. In contrast, preprocessing techniques such as those from Bai et al.^159^ and Zamir et al.^153^ show reduced effectiveness, particularly for SSD and Faster R-CNN, which suffer from substantial drops in precision. While YOLOv7 and YOLOv8 benefit moderately from enhancement techniques like CLAHE and H. Nguyen et al.^152^, their results remain inconsistent under moderate overexposure. These patterns highlight the critical role of selecting appropriate preprocessing methods tailored to both the lighting condition and model architecture. The results reaffirm that lightweight and modern architectures like YOLOv10 can maintain high detection accuracy even under visual degradation when supported by effective preprocessing strategies.

Detection accuracy under severe overexposure (Brightness2 condition) reveals critical differences in model resilience and preprocessing method effectiveness. As summarized in Table 7, YOLOv10 achieves the strongest average performance under Brightness 2 conditions when enhanced with techniques such as Afifi et al.^43^(0.57), H. Wang et al.^151^(0.515), and H. Nguyen et al.^152^(0.426), highlighting its robustness to lighting distortion when paired with suitable preprocessing. Afifi et al.^43^’s method proves to be the most adaptable across architectures, boosting performance not only in YOLOv10 but also in YOLOv8 (0.51) and YOLOv7 (0.498). In contrast, SSD and Faster R-CNN remain the most vulnerable under extreme brightness, with scores typically below 0.32 even after enhancement. Methods like Zamir et al.^153^’s lowlight adjustment show minimal benefit—or even degradation—under these conditions, reinforcing the importance of tailoring preprocessing strategies to specific lighting distortions. These results underscore that effective preprocessing, aligned with model architecture, plays a vital role in preserving detection performance under high-exposure scenarios.

Under moderately dark conditions (Darkness1), the impact of different preprocessing techniques on detection precision becomes more pronounced, especially when combined with modern object detectors. As shown in Table 8, enhancement methods like CLAHE and histogram equalization significantly improve precision across most YOLO variants. Notably, YOLOv10 paired with Afifi et al.^43^’s method achieves the highest score (0.783), while YOLOv7 combined with Zamir et al.^153^’s lowlight enhancement reaches an impressive 0.810—the highest among all entries. Traditional detectors like Faster R-CNN and SSD also benefit from preprocessing, particularly with histogram-based and contrast enhancement techniques. These findings emphasize that targeted preprocessing pipelines can effectively compensate for moderate underexposure, especially when tailored to the strengths of specific model architectures. The synergy between enhancement methods and modern YOLO versions highlights the potential of preprocessing as a robust solution for improving model resilience in low-light scenarios.

Severe underexposure introduces significant obstacles for object detection, particularly in safety-critical applications where reliable performance is non-negotiable. As shown in Table 9, precision scores under Darkness2 conditions reveal that advanced YOLO models—most notably YOLOv10—demonstrate notable robustness when complemented by effective preprocessing techniques. Methods developed by Bai et al.^159^, C. Guo et al.^160^, and Zamir et al.^153^(lowlight enhancement) consistently improve performance, with YOLOv10 reaching the highest recorded precision of 0.804 when paired with Bai et al.^159^’s enhancement approach. Earlier YOLO versions, such as YOLOv7, also show substantial gains under similar preprocessing. In contrast, default configurations lead to sharp performance degradation across all models, with SSD and Faster R-CNN exhibiting particularly weak resilience. These results underscore the critical importance of customized preprocessing strategies in restoring image quality and sustaining detection reliability under extreme low-light conditions.

The comparative analysis in Tables 5, 6, 7, 8 and 9 underscores how lighting conditions significantly affect object detection accuracy and the value of preprocessing techniques in mitigating these effects. Under normal lighting, the benefit of preprocessing is relatively modest. For example, YOLOv9 and YOLOv10 show less than 16% improvement in accuracy when enhanced by methods such as those from Afifi et al.^43^ and Cui et al.^158^, indicating that default performance is already strong and enhancements offer only incremental gains.

In contrast, extreme brightness and darkness reveal the true impact of preprocessing. Under Brightness2, YOLOv7’s performance improves by over 72% with Afifi et al.^43^’s method. Likewise, in low-light Darkness2 conditions, preprocessing yields dramatic gains—YOLOv5’s accuracy leaps from 0.24 to 0.726 with histogram equalization. YOLOv10 and Faster R-CNN perform exceptionally well when enhanced by methods like Bai et al.^159^, achieving top scores of 0.804 and 0.660, respectively. These findings confirm that while preprocessing may be optional in ideal conditions, it becomes essential for maintaining reliable detection performance in adverse lighting, especially for real-time applications where both robustness and speed are critical.

Given YOLOv10 superior performance across all conditions, we conducted an in-depth analysis of preprocessing effectiveness for this model. Table 10 presents the complete performance gains (mAP@0.5) relative to baseline for all 13 preprocessing methods tested. The “Default” column shows the baseline mAP@0.5 values without any preprocessing, while all other columns indicate the percentage improvement (positive values) or degradation (negative values) relative to these baseline values.

The analysis reveals clear preprocessing strategies for different lighting conditions. Under normal lighting (baseline precision = 0.678), Cui et al.^158^ method achieves the highest improvement at + 15.93%, though YOLOv10 strong baseline performance means preprocessing offers modest gains overall. For moderate overexposure (Brightness1, baseline = 0.466), H. Nguyen et al.^152^(+ 39.91%) and Nsampi et al.^161^(+ 38.63%) demonstrate exceptional effectiveness, while Zamir et al.^153^ lowlight enhancement counterproductively reduces performance by −71.03%. Severe overexposure (Brightness2, baseline = 0.375) presents the most challenging bright conditions, where Afifi et al.^43^ (+ 52.00%) and H. Wang et al.^151^(+ 37.33%) provide crucial recovery from the 44.7% performance drop from normal conditions.

In underexposed conditions, the pattern shifts dramatically. Moderate darkness (Darkness1, baseline = 0.662) maintains reasonable performance with modest preprocessing gains, with Afifi et al.^43^ leading at + 18.28%. However, severe underexposure (Darkness2, baseline = 0.198) reveals remarkable preprocessing effectiveness, with all methods achieving gains between + 184.34% and + 306.06%. The dramatic improvement from the critically low baseline (0.198) demonstrates that preprocessing is essential rather than optional in extreme darkness, with Bai et al.^159^(+ 306.06%), H. Wang et al.^151^(+ 303.54%), and C. Guo et al.^160^(+ 298.49%) effectively quadrupling the detection performance, transforming YOLOv10 from nearly non-functional to operationally viable in severe darkness conditions.

**Fig. 10:**
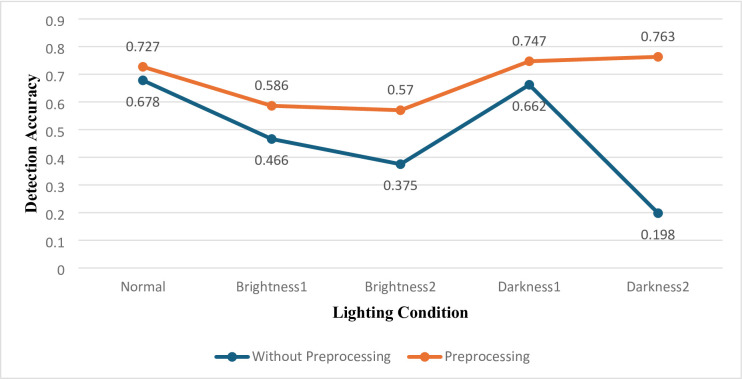
Visual Comparison of YOLOv10 Performance: With and Without Preprocessing. The “Preprocessing” line shows YOLOv10’s precision when paired with the preprocessing method that achieved the highest mean precision across all evaluated detectors (YOLOv5-v12, SSD, Faster R-CNN) for each lighting category: Histogram Equalisation for Normal, Afifi et al. for Brightness 1 and 2, and Zamir et al. (Lowlight Enhancement) for Darkness 1 and 2.

Figure 10 presents a graph comparing object detection results obtained using YOLOv10, our best-performing YOLO model, on challenging images captured under various lighting conditions. The graph illustrates a consistent performance improvement when preprocessing is applied, particularly in adverse lighting scenarios. While the accuracy gain is modest under normal conditions, it becomes significantly pronounced in Brightness2 and Darkness2 cases, where preprocessing boosts accuracy from 0.375 to 0.57 and from 0.198 to 0.763, respectively. These observations highlight the critical importance of preprocessing strategies in maintaining YOLOv10 detection reliability, especially under severe illumination distortions.

**Fig. 11:**
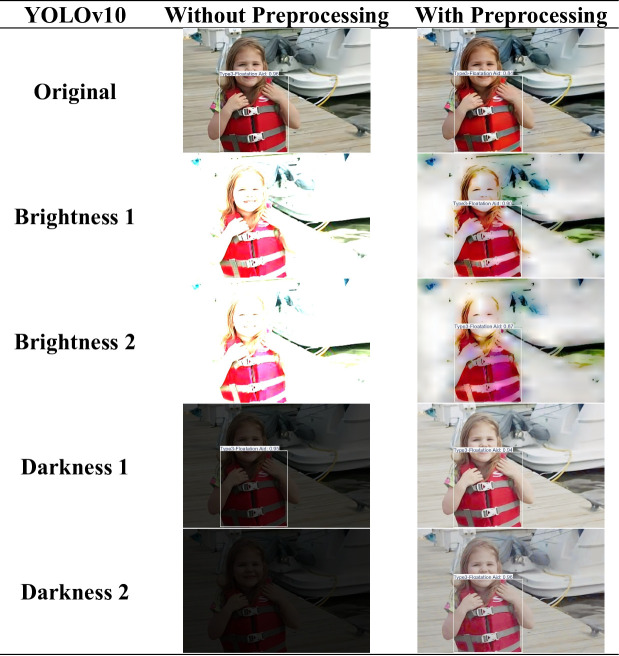
Visual Comparison Between Preprocessing and Without Preprocessing in The Best YOLO Version. Sample images from “Life Jacket” by CONTIL, available at https://universe.roboflow.com/contil-rn9fi/life-jacket-kt521 under CC BY 4.0 (https://creativecommons.org/licenses/by/4.0/). Images were cropped, resized, and enhanced for quality.

In addition to the performance trends illustrated in Fig. 10, a qualitative evaluation was conducted to observe how preprocessing affects object detection under challenging lighting scenarios. Figure 11 visually contrasts the output of YOLOv10—the best-performing model—on five test cases: one with normal lighting and four with varying degrees of overexposure and underexposure. Each row compares detection outcomes without preprocessing against results after applying enhancement techniques. The preprocessing methods include^43^ for brightened images and^153^ for darkened images. The improvements are evident in terms of clearer object boundaries, reduced false positives, and more accurate bounding boxes, especially in extreme lighting conditions. This comparison highlights how visual degradation caused by lighting can be effectively mitigated through appropriate enhancement strategies.

Overall, the extensive evaluation of multiple YOLO variants, SSD, and Faster R-CNN across five distinct lighting conditions reveals that preprocessing plays a pivotal role in enhancing object detection robustness, especially under extreme brightness or darkness. While preprocessing offers marginal benefits under normal conditions, its impact becomes substantial when visual input quality is compromised. YOLOv10 consistently demonstrates the highest adaptability and performance across scenarios, reaffirming its suitability for real-time, safety-critical applications. The combined quantitative and qualitative analyses confirm that strategically selected preprocessing pipelines not only recover lost visual fidelity but also substantially elevate detection accuracy, making them indispensable in deployment pipelines where environmental lighting is variable and uncontrollable.

## Result discussion

This section interprets the experimental results to answer three questions: (i) which detector and preprocessing combinations are most effective on this dataset; (ii) why do different preprocessing methods dominate under different lighting failure modes; and (iii) what do these findings imply specifically for maritime SAR deployment, beyond benchmark performance. We then identify the limitations of the present study and outline a path to operational validation.

### Detector performance and the YOLOv10–baseline comparison

YOLOv10 achieved the highest precision across all evaluated detectors at this dataset scale (*n* = 1,630), with a peak precision of 0.804 under preprocessed Darkness2 conditions and consistently strong performance across the five lighting variants reported in Tables 4, 5, 6, 7, 8, 9, 10, . We note that individual cells of these tables show other detectors (notably YOLOv8 with Afifi preprocessing under original lighting, and YOLOv7 with CLAHE under Darkness 1) achieving comparable or marginally higher peak precision under specific conditions; YOLOv10’s claim to the leading position rests on its consistent ranking near the top across all conditions combined with its real-time inference speed (43.9 FPS), not on dominance in every cell.

Conservative comparison with SSD and Faster R-CNN. The two non-YOLO baselines were trained from COCO-pretrained checkpoints, while all YOLO versions were trained from random initialisation (Sect. “Baseline detector configurations (SSD and faster R-CNN)”). YOLOv10’s precision advantage over the baselines therefore holds *despite* the baselines’ initialisation handicap on YOLO; if all detectors were trained from COCO weights, the gap would likely narrow (and could in some metrics reverse). We do not interpret our results as evidence that YOLOv10 is universally superior to two-stage detectors — only that, at the matched ~ 85,800–91,800-step training budget on this specific dataset, YOLOv10 offers the best precision–latency trade-off.

No claim about specific architectural causes. We do not claim that any single architectural component of YOLOv10 (NMS-free training, lightweight classification heads, rank-guided block design, etc.) is the cause of its observed advantage on our dataset, as we did not conduct the ablation studies that would be required to support such a claim. Future work could disentangle these factors through controlled ablations on the same dataset.

### Why different preprocessing methods dominate under different lighting failure modes

The preprocessing experiments revealed a striking dissociation: Afifi et al.‘s exposure correction^43^ was the single most effective method under overexposed conditions (Brightness 1 and Brightness 2), while Bai et al.‘s RetinexMamba^159^ was the most effective under severe underexposure (Darkness 2). For YOLOv10 specifically — the strongest detector overall — Afifi et al. raised precision from 0.375 (no preprocessing) to 0.570 under Brightness 2 — a + 52% relative improvement at the F1-optimal operating point. Bootstrap analysis on the threshold-independent mAP@0.5 metric gives a corresponding lift from 0.209 [0.155, 0.289] to 0.286 [0.225, 0.377], a + 37% relative improvement (Table 11). Under Darkness 2, the contrast was even more dramatic: precision rose from 0.198 (no preprocessing) to 0.804 with Bai et al. — a + 306% relative improvement at the F1-optimal operating point. Bootstrap analysis on mAP@0.5 gives a corresponding lift from 0.219 [0.161, 0.308] to 0.709 [0.636, 0.815], a + 224% relative improvement (Table 11) — both metrics confirm a substantial recovery that effectively transformed YOLOv10 from “nearly non-functional” to “operationally viable” in severe darkness. While Bai et al.’s method achieves YOLOv10’s highest single-cell precision under Darkness 2 (0.804), the broader cross-detector analysis underlying Fig. 10 identifies Zamir et al.’s Lowlight Enhancement as the most consistently effective preprocessing for darkness conditions across the full ensemble of evaluated detectors (YOLOv5-v12, SSD, Faster R-CNN), yielding 0.763 for YOLOv10 (+ 285%) with robust performance across the detector ensemble. Both perspectives have operational value: Bai et al.’s method for YOLOv10-specific peak performance, and Zamir et al.’s Lowlight Enhancement for detector-agnostic robustness in deployment. No single preprocessing method dominated across all lighting variants. We interpret this as follows.

Afifi et al. is well matched to the failure mode of overexposure because its training objective — paired correction of *both* overexposed and underexposed images using a single coarse-to-fine network — explicitly models the recovery of saturated highlights. On overexposed maritime images, the dominant failure mode is loss of contrast in the bright regions where life jackets reside (white spray against a bright sky, light-coloured jackets washed out by glare). Afifi et al.‘s coarse-to-fine residual prediction excels here because it allows controlled attenuation of saturated regions while preserving texture in mid-tones.

Bai et al. (RetinexMamba) is well matched to severe underexposure because the Retinex decomposition explicitly separates illumination from reflectance, and the Mamba-based state-space backbone allows efficient long-range reasoning across the image — necessary when very few pixels carry information and the model must propagate context from sparse highlights. On Darkness 2 images, the dominant failure mode is not just low brightness but signal-to-noise collapse in shadow regions where life jacket boundaries should be. Methods that only adjust brightness (histogram equalisation, gamma correction, CLAHE) amplify the noise along with the signal; Retinex-based decomposition with a learned reflectance prior recovers the signal more selectively.

Method mismatch is costly. Applying Bai et al. (a low-light specialist) to overexposed images *degraded* performance relative to no preprocessing in our experiments (relative change of approximately − 55% under Brightness 2 for YOLOv10), and conversely applying Afifi et al. to severe darkness was less effective than RetinexMamba. This dissociation has direct practical implications, discussed next.

### Implications for maritime SAR deployment

The dissociation between overexposure-suited and underexposure-suited preprocessing methods means that a single fixed preprocessing pipeline is unlikely to be optimal for SAR deployment, where lighting conditions vary continuously over a single search mission. We see three implications.

- Adaptive preprocessing dispatch.

A practical deployment should incorporate a lightweight scene-classifier that estimates whether an incoming frame is in the overexposed, underexposed, or near-normal regime, and dispatches the input to the appropriate preprocessing branch (e.g., Afifi et al. for overexposure, RetinexMamba for darkness, identity for normal lighting). The classifier itself can be implemented as a sub-1ms histogram-feature decision rule and does not add meaningful latency. The detection backbone (YOLOv10) remains shared across branches.

- Confidence calibration under preprocessing.

Preprocessing changes the input distribution to the detector relative to the (un-preprocessed) training distribution. In our results this manifest as detection-confidence shifts even when correctness is preserved. For SAR, where false negatives are far more costly than false positives, confidence thresholds should be re-calibrated per preprocessing branch using held-out validation data. This is a known issue with test-time input transformations and is not specific to our setting.

- Operational thresholds, not maximum precision.

A SAR system optimises for recall under acceptable false-positive load rather than precision in isolation. Our reported precision of 0.804 under preprocessed Darkness 2 is encouraging, but it is the recall column that determines whether the system would miss a person in distress. We report all four metrics (precision, recall, mAP@0.5, mAP@0.5:0.95) for the headline conditions in Table 3 (overall summary) and Table 4 (per-lighting baseline without preprocessing); precision across all lighting × preprocessing × detector combinations is reported in Tables 5, 6, 7, 8 , 9 and 10; mAP@0.5/mAP@0.5:0.95 with bootstrap confidence intervals for the YOLOv10 headline cells is reported in Table 11, to support this analysis, and we emphasise that operational threshold selection should weight recall heavily in any SAR deployment derived from this work.

### Limitations and the gap to operational deployment

We identify five limitations that bound the operational claims that can be drawn from this study, and that define the most important directions for follow-on work.

- Synthetic illumination perturbation.

Our five lighting conditions are generated by deterministic intensity scaling of the original test images. This does not reproduce the photographic phenomena of true overexposure or true low-light photography, which involve sensor noise that scales with exposure, lens flare, white-balance shifts, and motion blur from longer exposure times. Methods that perform well under our synthetic Darkness 2 may perform differently on genuine nighttime maritime imagery. Validation on a real low-light maritime dataset is a priority.

- Single-author annotation and dataset biases.

All 1,630 images were annotated by a single author, and the dataset was assembled from web image search results. This produces well-known biases — over-representation of clearly visible, single-target, daylight, fixed-viewpoint imagery — that will not generalise to all operational settings. Multi-annotator agreement studies and curated capture under genuine maritime conditions are required before any operational deployment.

- No multi-target or occluded subjects.

Real SAR scenes routinely contain multiple subjects in close proximity, partially submerged victims, and victims partially occluded by waves, debris, or vessel structures. Our dataset contains essentially none of these, which is the most consequential dataset gap for deployment readiness.

- Single training run per detector.

Compute constraints precluded multi-seed retraining; we partially address this through 95% bootstrap test-set confidence intervals on mAP@0.5 and mAP@0.5:0.95 (Sect. “Evaluation protocol”, Table 11), but the resulting uncertainty estimates do not capture training-induced variability. The absolute precision numbers reported in Tables 4, 5, 6, 7, 8, 9 and 10 should therefore be interpreted as point estimates for the single training run we performed, with bootstrap CIs on mAP-based metrics reflecting test-set sampling variability only.

- Fixed-viewpoint imagery.

All images are from broadly fixed (eye-level or shoulder-level) viewpoints. Aerial, vessel-deck, and water-surface viewpoints will produce different scale and aspect distributions, and would likely require either fine-tuning on viewpoint-matched data or geometric augmentation to preserve detection performance.

### Path to deployment

Subject to addressing the limitations above, the proposed pipeline could in principle support SAR teams as a decision-support tool. We outline a phased deployment pathway. Phase 1 (proof-of-concept): integration with edge-computing devices (NVIDIA Jetson AGX Orin or equivalent), achieving 30 + FPS at 320 × 320 input, with the system acting only as an alerting tool that flags potential detections for human verification. Phase 2 (sensor fusion): combine the visible-light pipeline with infrared and radar inputs to provide redundancy under adverse weather and at night, when the visible-light channel is least reliable. Phase 3 (semi-autonomous operation): introduce temporal-consistency checks across video frames, adaptive confidence thresholds tied to environmental sensors, and explainable-AI components (Grad-CAM, SHAP) to support operator trust and regulatory compliance.

Throughout all phases, we anticipate that the system will function alongside human operators, not autonomously. The cost of a missed detection in SAR — a life lost — is not symmetric with the cost of a false alarm, and any operational deployment of computer vision in SAR must be designed around this asymmetry rather than around aggregate accuracy metrics.

## Conclusion and future works

This study evaluated eight YOLO versions (v5–v12) and two non-YOLO baselines for life jacket detection, in combination with twelve image preprocessing methods, across five simulated lighting conditions. Two findings stand out. First, on the combined criterion of mean precision under original lighting while maintaining ≥ 30 FPS on Tesla T4 GPU, YOLOv10 emerged as the best-performing detector (precision 0.678, inference speed 43.9 FPS at 320 × 320 input). We note that other detectors achieve higher precision in isolation under specific cells (Table 5), but only YOLOv10 simultaneously satisfies the speed constraint for time-sensitive SAR deployment. Second, preprocessing was decisive only under extreme illumination: under original lighting the gain from preprocessing was marginal, but under severe overexposure and underexposure the best-matched preprocessing methods recovered detection performance from near-failure to operationally meaningful levels — Afifi et al.‘s exposure correction proved most effective for overexposed conditions and Bai et al.‘s RetinexMamba most effective for darkness, while no single method dominated across all conditions.

Future work will address the limitations identified in this study along three axes: (i) dataset expansion to include true nighttime imagery, multi-target scenes, occluded subjects, and small/distant targets; (ii) evaluation under genuine maritime variability — wave disturbance, sea spray, weather degradation — rather than synthetically perturbed lighting; and (iii) translation from benchmark to deployment, including edge-device inference (Jetson AGX Orin and equivalents), integration with thermal or radar sensors for redundancy under adverse conditions, and explainable AI components (Grad-CAM, SHAP) to support human-in-the-loop SAR decision-making. We emphasise that the present results are obtained on a curated benchmark with synthetic illumination perturbation; substantial additional validation under realistic maritime conditions is required before any operational claim can be made.

## Supplementary Information

Below is the link to the electronic supplementary material.

Supplementary Material 1

## Data Availability

The sources for the utilized datasets can be accessed at https://github.com/MikeJ-97/Advanced-Detection-of-Life-Jackets-in-Maritime-Environments-Using-YOLO-Algorithms.git.

## Code availability

All training, evaluation, and image preprocessing scripts used in this study are publicly available in the same repository as the dataset: https://github.com/MikeJ-97/Advanced-Detection-of-Life-Jackets-in-Maritime-Environments-Using-YOLO-Algorithms.git. The repository contains Colab notebooks for YOLO training and validation, configuration files for the SSD and Faster R-CNN baselines, the dataset YAML specification, and the preprocessing scripts used to generate the brightness- and darkness-augmented test sets.

## References

[1] Ho, W. C., Shen, J. H., Liu, C. P. & Chen, Y. W. Research on Optimal Model of Maritime Search and Rescue Route for Rescue of Multiple Distress Targets. *J. Mar. Sci. Eng.* **10**, 460. 10.3390/JMSE10040460/S1 (2022).

[2] Otote, D. A. et al. A decision-making algorithm for maritime search and rescue plan.. *Sustainability* **11**, 2084. 10.3390/SU11072084 (2019).

[3] Lidbetter, T. Search and rescue in the face of uncertain threats. *Eur. J. Oper. Res.* **285**, 1153–1160. 10.1016/J.EJOR.2020.02.029 (2020).

[4] Zhang, Y., Yin, Y. & Shao, Z. An enhanced target detection algorithm for maritime search and rescue based on aerial images. *Remote Sens.* **15**, 4818. 10.3390/RS15194818 (2023).

[5] Pitman, S. J., Wright, M. & Hocken, R. An analysis of lifejacket wear, environmental factors, and casualty activity on marine accident fatality rates. *Saf. Sci.* **111**, 234–242. 10.1016/J.SSCI.2018.07.016 (2019).

[6] Kjaergaard, B., Danielsen, A. V., Simonsen, C. & Wiberg, S. A paramilitary retrieval team for accidental hypothermia. Insights gained from a simple classification with advanced treatment over 16 years in Denmark. *Resuscitation* **156**, 114–119. 10.1016/J.RESUSCITATION.2020.09.008 (2020). 32946984 10.1016/j.resuscitation.2020.09.008

[7] Malyszko, M. Fuzzy logic in selection of maritime search and rescue units. *Appl. Sci. (Basel)* **12**, 21. 10.3390/APP12010021 (2021).

[8] Medić, D., Bakota, M., Jelaska, I. & Škorput, P. Low contrast challenge and limitations of thermal drones in maritime search and rescue—Pilot study. *Drones* **8**, 76. 10.3390/DRONES8030076 (2024).

[9] Solberg, K. E. Safety and Emergency Response Associated with Cold Climate Marine Operations. (2020). https://www.uis.no

[10] Li, C. et al. YOLOv6: A Single-Stage Object Detection Framework for Industrial Applications. (2022). https://arxiv.org/abs/2209.02976v1

[11] Redmon, J., Divvala, S., Girshick, R. & Farhadi, A. You Only Look Once: Unified, Real-Time Object Detection. (2015). https://arxiv.org/abs/1506.02640

[12] Wang, A. et al. YOLOv10: Real-Time End-to-End Object Detection. (2024). https://arxiv.org/abs/2405.14458v1

[13] Miri Rekavandi, A. et al. A Guide to Image and Video based Small Object Detection using Deep Learning: Case Study of Maritime Surveillance. (2022). https://arxiv.org/abs/2207.12926v1

[14] Zhao, B. & Song, R. Enhancing two-stage object detection models via data-driven anchor box optimization in UAV-based maritime SAR.. *Sci. Rep.* **14**(1), 1–11. 10.1038/s41598-024-55570-z (2024). 38413792 10.1038/s41598-024-55570-z PMC10899653

[15] Cafarelli, D. et al. MOBDrone: a Drone Video Dataset for Man OverBoard Rescue. (2022). https://arxiv.org/abs/2203.07973

[16] Tallón-Ballesteros, A. J. & Beltrán-Barba, R. Fuzzy systems and data mining IX: proceedings of FSDM 2023. (2023). https://books.google.com/books/about/Fuzzy_Systems_and_Data_Mining_IX.html?hl=zh-CN&id=BYLyEAAAQBAJ

[17] Xu, J. et al. YoloOW: A spatial scale adaptive real-time object detection neural network for open water search and rescue from UAV aerial imagery.. *IEEE Trans. Geosci. Remote Sens.*10.1109/TGRS.2024.3395483 (2024).

[18] Emanuelsson, E. & Wang, L. Real-time characteristics of marine object detection under low light conditions Marine object detection using YOLO with near infrared camera Master’s thesis in Master Program Embedded Electronic system design. (2020). https://hdl.handle.net/20.500.12380/302142

[19] Ma, R. et al. Improved ship object detection in low-illumination environments using RetinaMFANet.. *J. Mar. Sci. Eng.* **10**, 1996. 10.3390/JMSE10121996 (2022).

[20] Sun, X., Liu, T., Yu, X. & Pang, B. Unmanned surface vessel visual object detection under all-weather conditions with optimized feature fusion network in YOLOv4. *J. Intell. Robot. Syst.: Theory Appl.* **103**, 1–16. 10.1007/S10846-021-01499-8/METRICS (2021).

[21] Varga, L. A., Kiefer, B., Messmer, M. & Zell, A. SeaDronesSee: A Maritime Benchmark for Detecting Humans in Open Water. (2021). https://arxiv.org/abs/2105.01922

[22] Wu, Z. et al. Delving into Robust Object Detection from Unmanned Aerial Vehicles: A Deep Nuisance Disentanglement Approach. In *Proceedings of the IEEE International Conference on Computer Vision*, 2019-October, 1201–1210 (2019). 10.1109/ICCV.2019.00129

[23] Charters, G., Cruz, S., Bernardino, A. & Cruz, G. Aerial Detection in Maritime Scenarios Using Convolutional Neural Networks. (2018). 10.1007/978-3-319-48680-2_33

[24] Jeong, C. Y., Yang, H. S. & Moon, K. Fast horizon detection in maritime images using region-of-interest.. *Int. J. Distrib. Sens. Netw.*10.1177/1550147718790753 (2018).

[25] Zhang, Y., Li, Q. Z. & Zang, F. N. Ship detection for visual maritime surveillance from non-stationary platforms. *Ocean Eng.* **141**, 53–63. 10.1016/J.OCEANENG.2017.06.022 (2017).

[26] Fu, Z., Xiao, Y., Tao, F., Si, P. & Zhu, L. DLSW-YOLOv8n: A novel small maritime search and rescue object detection framework for UAV images with deformable large kernel net. *Drones* **8**, 310. 10.3390/DRONES8070310 (2024).

[27] Huang, K. & Chong, W. ReIDTracker Sea: the technical report of BoaTrack and SeaDronesSee-MOT challenge at MaCVi of WACV24. (2023). https://arxiv.org/abs/2311.07616

[28] Rekavandi, A. M. et al. A Guide to Image and Video based Small Object Detection using Deep Learning: Case Study of Maritime Surveillance. (2022). https://arxiv.org/abs/2207.12926

[29] Yang, C. Y. et al. Sea You Later: Metadata-Guided Long-Term Re-Identification for UAV-Based Multi-Object Tracking. (2023). https://arxiv.org/abs/2311.03561

[30] Shen, D., Wu, G. & Suk, H. I. Deep learning in medical image analysis.. *Annu. Rev. Biomed. Eng.* **19**, 221–248. 10.1146/ANNUREV-BIOENG-071516-044442/CITE/REFWORKS (2017). 28301734 10.1146/annurev-bioeng-071516-044442 PMC5479722

[31] Kuutti, S., Bowden, R., Jin, Y., Barber, P. & Fallah, S. A Survey of Deep Learning Applications to Autonomous Vehicle Control. (2019). https://arxiv.org/abs/1912.10773

[32] Kraus, M., Feuerriegel, S. & Oztekin, A. Deep learning in business analytics and operations research: Models, applications and managerial implications. *Eur. J. Oper. Res.* **281**, 628–641. 10.1016/J.EJOR.2019.09.018 (2020).

[33] Balaban, S. Deep learning and face recognition: the state of the art. (2015). https://www.lambdalabs.com

[34] Hu, G. et al. When Face Recognition Meets with Deep Learning: an Evaluation of Convolutional Neural Networks for Face Recognition (2015).

[35] Chen, W. & Shah, T. Exploring Low-light Object Detection Techniques (2021).

[36] Karim, M. M. et al. A region-based deep learning algorithm for detecting and tracking objects in manufacturing plants. *Procedia Manuf.* **39**, 168–177. 10.1016/J.PROMFG.2020.01.289 (2019).

[37] Mukherjee, R., Bessa, M., Melo-Pinto, P. & Chalmers, A. Object detection under challenging lighting conditions using high dynamic range imagery. *IEEE Access* **9**, 77771–77783. 10.1109/ACCESS.2021.3082293 (2021).

[38] Wang, L., He, J. & Chen, J. Real-Time Object Detection in Overexposed Images Based on YOLOv5. (2024). 10.1109/ICETCI61221.2024.10594528

[39] Wu, Y. & Tsotsos, J. Active Control of Camera Parameters for Object Detection Algorithms. (2017). http://jtl.lassonde.yorku.ca/software/datasets/

[40] Xiao, Y., Jiang, A., Ye, J. & Wang, M. W. Making of night vision: Object detection under low-illumination. *IEEE Access* **8**, 123075–123086. 10.1109/ACCESS.2020.3007610 (2020).

[41] Xu, X., Wang, S., Wang, Z., Zhang, X. & Hu, R. Exploring Image Enhancement for Salient Object Detection in Low Light Images. (2020). 10.1145/nnnnnnn.nnnnnnn

[42] Zhou, H., Sun, M., Ren, X. & Wang, X. Visible-thermal image object detection via the combination of illumination conditions and temperature information. *Remote Sens.* **13**, 3656. 10.3390/RS13183656 (2021).

[43] Afifi, M., Derpanis, K. G., Ommer, B. & Brown, M. S. Learning Multi-Scale Photo Exposure Correction. (2020). https://arxiv.org/abs/2003.11596

[44] Ye, L., Wang, D., Yang, D., Ma, Z. & Zhang, Q. VELIE: A vehicle-based efficient low-light image enhancement method for intelligent vehicles. *Sensors* **24**, 1345. 10.3390/S24041345 (2024). 38400503 10.3390/s24041345 PMC10892397

[45] Chaudhuri, D. et al. A statistical approach for automatic detection of ocean disturbance features from SAR images. *IEEE J. Sel. Top. Appl. Earth Obs. Remote Sens.* **5**, 1231–1242. 10.1109/JSTARS.2012.2186630 (2012).

[46] Chen, M., Sun, J., Aida, K. & Takefusa, A. Weather-aware object detection method for maritime surveillance systems. *Future Gener. Comput. Syst.* **151**, 111–123. 10.1016/J.FUTURE.2023.09.030 (2024).

[47] Cheng, N., Xie, H., Zhu, X. & Wang, H. Joint image enhancement learning for marine object detection in natural scene. *Eng. Appl. Artif. Intell.* **120**, 105905. 10.1016/J.ENGAPPAI.2023.105905 (2023).

[48] Yang, Z., Li, Y., Wang, B., Ding, S. & Jiang, P. A lightweight sea surface object detection network for unmanned surface vehicles. *J. Mar. Sci. Eng.* **10**, 965. 10.3390/JMSE10070965 (2022).

[49] Redmon, J. & Farhadi, A. YOLO9000: Better, Faster, Stronger. (2016). http://pjreddie.com/yolo9000/

[50] Redmon, J. & Farhadi, A. YOLOv3: An Incremental Improvement. (2018). https://arxiv.org/abs/1804.02767v1

[51] Bochkovskiy, A., Wang, C. Y. & Liao, H. Y. M. YOLOv4: Optimal Speed and Accuracy of Object Detection. (2020). https://arxiv.org/abs/2004.10934v1

[52] Reis, D., Kupec, J., Hong, J. & Daoudi, A. Real-Time Flying Object Detection with YOLOv8. (2023). https://arxiv.org/abs/2305.09972v2

[53] Wang, C. Y., Bochkovskiy, A. & Liao, H. Y. M. YOLOv7: Trainable bag-of-freebies sets new state-of-the-art for real-time object detectors. (2022). https://arxiv.org/abs/2207.02696

[54] Wang, C. Y., Yeh, I. H. & Liao, H. Y. M. YOLOv9: Learning What You Want to Learn Using Programmable Gradient Information. (2024). https://arxiv.org/abs/2402.13616v2

[55] Zhu, X., Lyu, S., Wang, X. & Zhao, Q. TPH-YOLOv5: Improved YOLOv5 Based on Transformer Prediction Head for Object Detection on Drone-captured Scenarios. In *Proceedings of the IEEE International Conference on Computer Vision*, 2021-October, 2778–2788. 10.1109/ICCVW54120.2021.00312 (2021).

[56] Dollár, P. & Zitnick, C. L. Fast edge detection using structured forests. *IEEE Trans. Pattern Anal. Mach. Intell.* **37**, 1558–1570. 10.1109/TPAMI.2014.2377715 (2015). 26352995 10.1109/TPAMI.2014.2377715

[57] Lee, D., Kim, G., Kim, D., Myung, H. & Choi, H. T. Vision-based object detection and tracking for autonomous navigation of underwater robots. *Ocean Eng.* **48**, 59–68. 10.1016/J.OCEANENG.2012.04.006 (2012).

[58] Lucchese Yz, L. & Mitra, S. K. Color Image Segmentation: A State-of-the-Art Survey. (2001). https://www.ece.lsu.edu/gunturk/Topics/Segmentation-2.pdf

[59] Rizzini, D. L., Kallasi, F., Oleari, F. & Caselli, S. Investigation of vision-based underwater object detection with multiple datasets. *Int. J. Adv. Rob. Syst.*10.5772/60526/ASSET/IMAGES/LARGE/10.5772_60526-FIG13.JPEG (2015).

[60] Sharma, N., Mishra, M., & Shrivastava, M. Colour image segmentation techniques and issues: an approach. *Int. J. Sci. & Technol. Res.* **1**(4). (2012). www.ijstr.org

[61] Du, Q. et al. A lightweight man-overboard detection and tracking model using aerial images for maritime search and rescue. *Remote Sens.* **16**, 165. 10.3390/RS16010165 (2023).

[62] Lyu, Z. et al. Real-time ship detection system for wave glider based on YOLOv5s-lite-CBAM model. *Appl. Ocean Res.* **144**, 103833. 10.1016/J.APOR.2023.103833 (2024).

[63] Zhang, Y. et al. An intelligent self-powered life jacket system integrating multiple triboelectric fiber sensors for drowning rescue. *InfoMat* **6**, e12534. 10.1002/INF2.12534 (2024).

[64] He, G. et al. An improved YOLO v4 algorithm-based object detection method for maritime vessels. *Int. J. Sci. Eng. Appl.* **11**, 50–55. 10.7753/IJSEA1104.1001 (2022).

[65] Kim, J. H., Kim, N., Park, Y. W. & Won, C. S. Object detection and classification based on YOLO-V5 with improved maritime dataset. *J. Mar. Sci. Eng.* **10**, 377. 10.3390/JMSE10030377 (2022).

[66] Kapur, S. V., Marine search and rescue using light weight & neural networks. (2019). http://hdl.handle.net/10222/76281

[67] Kiefer, B., Ott, D. & Zell, A. Leveraging synthetic data in object detection on unmanned aerial vehicles. (2021). https://git.io/Jyf5j

[68] Algarni, A. D. Efficient object detection and classification of heat emitting objects from infrared images based on deep learning. *Multim. Tools Appl.* **79**, 13403–13426. 10.1007/S11042-020-08616-Z (2020).

[69] Gallagher, J. E. & Oughton, E. J. Assessing thermal imagery integration into object detection methods on air-based collection platforms. *Sci. Rep.* **13**(1), 1–12. 10.1038/s41598-023-34791-8 (2023). 37231037 10.1038/s41598-023-34791-8 PMC10212993

[70] Uzair, M., Brinkworth, R. S. A. & Finn, A. Detecting small size and minimal thermal signature targets in infrared imagery using biologically inspired vision. *Sensors* **21**, 1812. 10.3390/S21051812 (2021). 33807741 10.3390/s21051812 PMC7961815

[71] Ding, Y. et al. Long-distance vehicle dynamic detection and positioning based on Gm-APD Lidar and LIDAR-YOLO. *IEEE Sens. J.* **22**, 17113–17125. 10.1109/JSEN.2022.3193740 (2022).

[72] Herzog, M. & Dietmayer, K. Training a Fast Object Detector for LiDAR Range Images Using Labeled Data from Sensors with Higher Resolution. In *2019 IEEE Intelligent Transportation Systems Conference, ITSC 2019*, 2707–2713. 10.1109/ITSC.2019.8917011 (2019).

[73] Sualeh, M. & Kim, G. W. Dynamic multi-LiDAR based multiple object detection and tracking. *Sensors* **19**, 1474. 10.3390/S19061474 (2019). 30917566 10.3390/s19061474 PMC6470994

[74] Wu, Y., Wang, Y., Zhang, S. & Ogai, H. Deep 3D object detection networks using LiDAR data: A review. *IEEE Sens. J.* **21**, 1152–1171. 10.1109/JSEN.2020.3020626 (2021).

[75] Park, J. J., Park, K. A., Kim, T. S., Oh, S. & Lee, M. Aerial hyperspectral remote sensing detection for maritime search and surveillance of floating small objects. *Adv. Space Res.* **72**, 2118–2136. 10.1016/J.ASR.2023.06.055 (2023).

[76] Wang, K. et al. An Intelligent Life Jacket System Based on OneNET. In *2021 IEEE 6th International Conference on Intelligent Computing and Signal Processing, ICSP 2021*, 1071–1074. 10.1109/ICSP51882.2021.9408758 (2021).

[77] Nguyen, D. B., Le Minh, T., Van Truc, N. & Vu Tran, H. Building a Smart Life Jacket Based on the IoT Platform. In *International Conference on Advanced Technologies for Communications*, 527–533 (2023). 10.1109/ATC58710.2023.10318940

[78] Serra, A. A., Nepa, P. & Manara, G. A wearable multi antenna system on a life jacket for Cospas Sarsat rescue applications. In *IEEE Antennas and Propagation Society, AP-S International Symposium (Digest)*, 1319–1322. 10.1109/APS.2011.5996532 (2011).

[79] Nixon, M. S. & Aguado, A. S. Feature Extraction and Image Processing for Computer Vision. In *Feature Extraction and Image Processing for Computer Vision*, 1–609 (2012). 10.1016/C2011-0-06935-1

[80] Sonka, M., Hlavac, V. & Boyle, R. Image processing, analysis and machine vision. *Image Process. Anal. Mach. Vis.*10.1007/978-1-4899-3216-7 (1993).

[81] Szeliski, R. *Computer Vision* (2022). 10.1007/978-3-030-34372-9.

[82] Wiley, V. & Lucas, T. Computer vision and image processing: A paper review. *Int. J. Artif. Intell. Res.* **2**, 29–36 (2018).

[83] Cazzato, D., Cimarelli, C., Sanchez-Lopez, J. L., Voos, H. & Leo, M. A. Survey of computer vision methods for 2D object detection from unmanned aerial vehicles. *J. Imaging* **6**, 78. 10.3390/JIMAGING6080078 (2020). 34460693 10.3390/jimaging6080078 PMC8321148

[84] Foresti, G. L. & Gentili, S. A vision based system for object detection in underwater images. *Int. J. Pattern Recognit. Artif. Intell.* **14**(02), 167–188. 10.1142/S021800140000012X (2011).

[85] Jiao, L. et al. A survey of deep learning-based object detection. *IEEE Access.* **7**, 128837–128868. 10.1109/ACCESS.2019.2939201 (2019).

[86] Osep, A., Mehner, W., Voigtlaender, P. & Leibe, B. Track, then Decide: Category-Agnostic Vision-based Multi-Object Tracking. In *Proceedings - IEEE International Conference on Robotics and Automation*, 3494–3501. 10.1109/ICRA.2018.8460975 (2017).

[87] Rashid, E., Ansari, M. D., Gunjan, V. K. & Ahmed, M. Improvement in extended object tracking with the vision-based algorithm. *Stud. Comput. Intell.* **885**, 237–245. 10.1007/978-3-030-38445-6_18 (2020).

[88] Shahzad, A., Gao, X., Yasin, A., Javed, K. & Anwar, S. M. A vision-based path planning and object tracking framework for 6-DOF robotic manipulator. *IEEE Access.* **8**, 203158–203167. 10.1109/ACCESS.2020.3037540 (2020).

[89] Wu, Y., Sui, Y. & Wang, G. Vision-based real-time aerial object localization and tracking for UAV sensing system. *IEEE Access.* **5**, 23969–23978. 10.1109/ACCESS.2017.2764419 (2017).

[90] Adjabi, I., Ouahabi, A., Benzaoui, A. & Taleb-Ahmed, A. Past, present, and future of face recognition: A review. *Electronics* **9**, 1188. 10.3390/ELECTRONICS9081188 (2020).

[91] Cevikalp, H. & Triggs, B. Face recognition based on image sets. In *Proceedings of the IEEE Computer Society Conference on Computer Vision and Pattern Recognition*, 2567–2573 (2010). 10.1109/CVPR.2010.5539965

[92] Guo, G. & Zhang, N. A survey on deep learning based face recognition. *Comput. Vis. Image Underst.* **189**, 102805. 10.1016/J.CVIU.2019.102805 (2019).

[93] Parmar, D. N. & Mehta, B. B. Face Recognition Methods & Applications. (2014). https://arxiv.org/abs/1403.0485v1

[94] Kiefer, B., Quan, Y. & Zell, A. Memory Maps for Video Object Detection and Tracking on UAVs. (2023). https://arxiv.org/abs/2303.03508

[95] Ye, C. & Li, J. Life Jacket Wearing Recognition Based on Semantic Segmentation. In *Proceedings – 2022 9th International Conference on Digital Home, ICDH 2022*, 141–146 (2022). 10.1109/ICDH57206.2022.00029

[96] Benjumea, A., Teeti, I., Cuzzolin, F. & Bradley, A. YOLO-Z: Improving small object detection in YOLOv5 for autonomous vehicles. (2021). https://arxiv.org/abs/2112.11798v4

[97] Liang, S. et al. Edge YOLO: Real-time intelligent object detection system based on edge-cloud cooperation in autonomous vehicles. *IEEE Trans. Intell. Transp. Syst.* **23**, 25345–25360. 10.1109/TITS.2022.3158253 (2022).

[98] Jha, S., Seo, C., Yang, E. & Joshi, G. P. Real time object detection and trackingsystem for video surveillance system. *Multimed. Tools Appl.* **80**, 3981–3996. 10.1007/S11042-020-09749-X/METRICS (2021).

[99] Narejo, S., Pandey, B., Esenarro Vargas, D., Rodriguez, C. & Anjum, M. R. Weapon detection using YOLO V3 for smart surveillance system. *Math. Probl. Eng.* **2021**, 9975700. 10.1155/2021/9975700 (2021).

[100] Ren, S., He, K., Girshick, R. & Sun, J. Faster R-CNN: Towards real-time object detection with region proposal networks. *IEEE Trans. Pattern Anal. Mach. Intell.* **39**, 1137–1149. 10.1109/TPAMI.2016.2577031 (2015). 10.1109/TPAMI.2016.2577031 27295650

[101] Liu, Y., Ma, Z., Liu, X., Ma, S. & Ren, K. Privacy-preserving object detection for medical images with Faster R-CNN. *IEEE Trans. Inf. Forensics Secur.* **17**, 69–84. 10.1109/TIFS.2019.2946476 (2022).

[102] Xu, J., Ren, H., Cai, S. & Zhang, X. An improved faster R-CNN algorithm for assisted detection of lung nodules. *Comput. Biol. Med.* **153**, 106470. 10.1016/J.COMPBIOMED.2022.106470 (2023). 36587571 10.1016/j.compbiomed.2022.106470

[103] Cao, Y., Cheng, X., Mu, J., Li, D. & Han, F. Detection method based on image enhancement and an improved faster R-CNN for failed satellite components. *IEEE Trans. Instrum. Meas.*10.1109/TIM.2023.3237809 (2023).

[104] Zhao, K., Kang, J., Jung, J. & Sohn, G. Building extraction from satellite images using mask R-CNN with building boundary regularization. In *IEEE Computer Society Conference on Computer Vision and Pattern Recognition Workshops*, 2018-June, 242–246 (2018). 10.1109/CVPRW.2018.00045

[105] Liu, W. et al. SSD: Single Shot MultiBox Detector. In *Lecture Notes in Computer Science (Including Subseries Lecture Notes in Artificial Intelligence and Lecture Notes in Bioinformatics)* 9905 LNCS, 21–37 (2015). 10.1007/978-3-319-46448-0_2

[106] Chandan, G., Jain, A. & Jain, H. Real Time Object Detection and Tracking Using Deep Learning and OpenCV. In *Proceedings of the International Conference on Inventive Research in Computing Applications, ICIRCA 2018*, 1305–1308 (2018). 10.1109/ICIRCA.2018.8597266

[107] Rohan, A., Rabah, M. & Kim, S. H. Convolutional neural network-based real-time object detection and tracking for Parrot AR Drone 2. *IEEE Access* **7**, 69575–69584. 10.1109/ACCESS.2019.2919332 (2019).

[108] Deng, J. et al. Single shot video object detector. *IEEE Trans. Multimed.* **23**, 846–858. 10.1109/TMM.2020.2990070 (2020).

[109] Thakar, V. et al. Efficient Single-Shot Multibox Detector for Construction Site Monitoring. In *IEEE International Smart Cities Conference, ISC2 2018* (2018). 10.1109/ISC2.2018.8656929

[110] Ibrahim, H. & Kong, N. S. P. Brightness preserving dynamic histogram equalization for image contrast enhancement. *IEEE Trans. Consum. Electron.* **53**, 1752–1758. 10.1109/TCE.2007.4429280 (2007).

[111] Park, G. H., Cho, H. H. & Choi, M. R. A contrast enhancement method using dynamic range separate histogram equalization. *IEEE Trans. Consum. Electron.* **54**, 1981–1987. 10.1109/TCE.2008.4711262 (2008).

[112] Pizer, S. M. et al. Adaptive histogram equalization and its variations. *Comput. Vision Graph. Image Process.* **39**, 355–368. 10.1016/S0734-189X(87)80186-X (1987).

[113] Stark, J. A. Adaptive image contrast enhancement using generalizations of histogram equalization. *IEEE Trans. Image Process.* **9**, 889–896. 10.1109/83.841534 (2000). 18255459 10.1109/83.841534

[114] Wang, C. & Ye, Z. Brightness preserving histogram equalization with maximum entropy: A variational perspective. *IEEE Trans. Consum. Electron.* **51**, 1326–1334. 10.1109/TCE.2005.1561863 (2005).

[115] Akila, K., Jayashree, L. S. & Vasuki, A. Mammographic image enhancement using indirect contrast enhancement techniques: A comparative study. *Procedia Comput. Sci.* **47**(C), 255–261. 10.1016/J.PROCS.2015.03.205 (2015).

[116] Patel, O., Maravi, Y. P. S. & Sharma, S. A comparative study of histogram equalization based image enhancement techniques for brightness preservation and contrast enhancement. *Signal Image Process Int J* **4**, 11–25. 10.5121/sipij.2013.4502 (2013).

[117] Zhang, W. et al. Underwater image enhancement via minimal color loss and locally adaptive contrast enhancement. *IEEE Trans. Image Process.* **31**, 3997–4010. 10.1109/TIP.2022.3177129 (2022). 10.1109/TIP.2022.3177129 35657839

[118] Chai, H. Y., Swee, T. T., Seng, G. H. & Wee, L. K. Multipurpose contrast enhancement on epiphyseal plates and ossification centers for bone age assessment. **12** (1) (2013). https://www.scopus.com/inward/record.uri?eid=2-s2.0-84875846033&doi=10.1186%2f1475-925X-12-27&partnerID=40&md5=7f0f052d3ea4f2668768e5de90344493 10.1186/1475-925X-12-27 PMC3652796 23565999

[119] Hossain, M. B. et al. Contrast enhancement of ultrasound imaging of the knee joint cartilage for early detection of knee osteoarthritis. **13**(1), 157–167 (2014). https://www.scopus.com/inward/record.uri?eid=2-s2.0-84901351091&doi=10.1016%2fj.bspc.2014.04.008&partnerID=40&md5=e21d61da057f55f84e2af049d12e870d

[120] Hum, Y. C. et al. A contrast enhancement framework under uncontrolled environments based on just noticeable difference. **103** (2022). https://www.scopus.com/inward/record.uri?eid=2-s2.0-85126081509&doi=10.1016%2fj.image.2022.116657&partnerID=40&md5=4fdbd7a6a5ab4dd63d13a9381f728f1c

[121] Singh, T. R., Roy, S., Singh, O. I., Sinam, T. & Singh Kh. M. A New Local Adaptive Thresholding Technique in Binarization. (2012). https://arxiv.org/abs/1201.5227v1

[122] Eilertsen, G., Kronander, J., Denes, G., Mantiuk, R. K. & Unger, J. HDR image reconstruction from a single exposure using deep CNNs. *ACM Trans. Graph.* **36**(6), 1. 10.1145/3130800.3130816 (2017).

[123] Wang, L. & Yoon, K. J. Deep learning for HDR imaging: State-of-the-art and future trends. *IEEE Trans. Pattern Anal. Mach. Intell.* **44**, 8874–8895. 10.1109/TPAMI.2021.3123686 (2022). 34714739 10.1109/TPAMI.2021.3123686

[124] Matsushita, Y., Ge, W., Tang, X. & Shum, H. Y. Full-frame video stabilization with motion inpainting. *IEEE Trans. Pattern Anal. Mach. Intell.* **28**, 1150–1163. 10.1109/TPAMI.2006.141 (2006). 16792103 10.1109/TPAMI.2006.141

[125] Rodriguez-Gomez, J. P., de Dios, J. R. M., Ollero, A. & Gallego, G. On the benefits of visual stabilization for frame- and event-based perception. *IEEE Robot. Autom. Lett.*10.1109/LRA.2024.3450290 (2024).

[126] Yang, D. et al. A streamlined approach for intelligent ship object detection using EL-YOLO algorithm. *Sci. Rep.* **14**(1), 1–17. 10.1038/s41598-024-64225-y (2024). 38956185 10.1038/s41598-024-64225-y PMC11219897

[127] Jin, Z. et al. Mining Contextual Information Beyond Image for Semantic Segmentation. In *Proceedings of the IEEE International Conference on Computer Vision*, 7211–7221 (2021). 10.1109/ICCV48922.2021.00714

[128] Li, J. et al. Attentive contexts for object detection. *IEEE Trans. Multimed.* **19**, 944–954. 10.1109/TMM.2016.2642789 (2017).

[129] Agarwal, S., Awan, A. & Roth, D. Learning to detect objects in images via a sparse, part-based representation. *IEEE Trans. Pattern Anal. Mach. Intell.* **26**, 1475–1490. 10.1109/TPAMI.2004.108 (2004). 15521495 10.1109/TPAMI.2004.108

[130] Mordan, T., Thome, N., Cord, M. & Henaff, G. Deformable Part-based Fully Convolutional Network for Object Detection. In *British Machine Vision Conference* BMVC 2017 (2017). 10.5244/c.31.88

[131] Zhang, N., Donahue, J., Girshick, R., Darrell, T. Part-Based, R-C-N-N. for Fine-Grained Category Detection. In *Lecture Notes in Computer Science (Including Subseries Lecture Notes in Artificial Intelligence and Lecture Notes in Bioinformatics)*, 8689 LNCS(PART 1), 834–849 (2014). 10.1007/978-3-319-10590-1_54

[132] Bolya, D., Zhou, C., Xiao, F. & Lee, Y. J. YOLACT: Real-time Instance Segmentation. (2019). https://arxiv.org/abs/1904.02689v2 10.1109/TPAMI.2020.3014297 32755851

[133] Hafiz, A. M. & Bhat, G. M. A survey on instance segmentation: State of the art. *Int. J. Multimed. Inf. Retr.* **9**, 171–189. 10.1007/S13735-020-00195-X/METRICS (2020).

[134] Li, Y. et al. YOLO-ACN: Focusing on small target and occluded object detection. *IEEE Access* **8**, 227288–227303. 10.1109/ACCESS.2020.3046515 (2020).

[135] Wang, A., Sun, Y., Kortylewski, A. & Yuille, A. Robust Object Detection under Occlusion with Context-Aware Compositional Nets. In *Proceedings of the IEEE Computer Society Conference on Computer Vision and Pattern Recognition*, 12642–12651 (2020). 10.1109/CVPR42600.2020.01266

[136] Xiao, M. et al. TDAPNet: Prototype Network with Recurrent Top-Down Attention for Robust Object Classification under Partial Occlusion. In *Lecture Notes in Computer Science (Including Subseries Lecture Notes in Artificial Intelligence and Lecture Notes in Bioinformatics)* 12536 LNCS, 447–463 (2019). 10.1007/978-3-030-66096-3_31

[137] Farid, H. Blind inverse gamma correction. *IEEE Trans. Image Process.* **10**, 1428–1433. 10.1109/83.951529 (2001). 18255487 10.1109/83.951529

[138] Maini, R. & Aggarwal, H. A. Comprehensive Review of Image Enhancement Techniques. (2010). https://arxiv.org/abs/1003.4053

[139] Grossberg, M. D. & Nayar, S. K. Determining the camera response from images: What is knowable?. *IEEE Trans. Pattern Anal. Mach. Intell.* **25**, 1455–1467. 10.1109/TPAMI.2003.1240119 (2003).

[140] Reza, A. M. Realization of the contrast limited adaptive histogram equalization (CLAHE) for real-time image enhancement. *J. VLSI Signal Process. Syst. Signal Image Video Technol.* **38**, 35–44. 10.1023/B:VLSI.0000028532.53893.82/METRICS (2004).

[141] Fu, X. et al. A retinex-based enhancing approach for single underwater image. In *IEEE International Conference on Image Processing, ICIP 2014*, 4572–4576 (2014). 10.1109/ICIP.2014.7025927

[142] Guo, X., Li, Y. & Ling, H. LIME: Low-light image enhancement via illumination map estimation. *IEEE Trans. Image Process.* **26**, 982–993. 10.1109/TIP.2016.2639450 (2017). 28113318 10.1109/TIP.2016.2639450

[143] Li, M., Liu, J., Yang, W., Sun, X. & Guo, Z. Structure-revealing low-light image enhancement via robust retinex model. *IEEE Trans. Image Process.* **27**, 2828–2841. 10.1109/TIP.2018.2810539 (2018). 29570085 10.1109/TIP.2018.2810539

[144] Parihar, A. S. & Singh, K. A study on Retinex based method for image enhancement. In *Proceedings of the 2nd International Conference on Inventive Systems and Control, ICISC 2018*, 619–624 (2018). 10.1109/ICISC.2018.8398874

[145] Wang, S., Zheng, J., Hu, H. M. & Li, B. Naturalness preserved enhancement algorithm for non-uniform illumination images. *IEEE Trans. Image Process.* **22**, 3538–3548. 10.1109/TIP.2013.2261309 (2013). 23661319 10.1109/TIP.2013.2261309

[146] Xu, J. et al. STAR: A structure and texture aware Retinex model. *IEEE Trans. Image Process.* **29**, 5022–5037. 10.1109/TIP.2020.2974060 (2020). 10.1109/TIP.2020.2974060 32167892

[147] Ignatov, A. et al. DSLR-Quality Photos on Mobile Devices with Deep Convolutional Networks. In *Proceedings of the IEEE International Conference on Computer Vision*, 2017-October, 3297–3305 (2017). 10.1109/ICCV.2017.355

[148] Wei, C., Wang, W., Yang, W. & Liu, J. Deep Retinex Decomposition for Low-Light Enhancement. (2018). https://arxiv.org/abs/1808.04560v1

[149] Chen, C., Chen, Q., Xu, J. & Koltun, V. Learning to See in the Dark. In *Proceedings of the IEEE Computer Society Conference on Computer Vision and Pattern Recognition*, 3291–3300 (2018). 10.1109/CVPR.2018.00347

[150] Zhang, Y., Zhang, J. & Guo, X. Kindling the Darkness: A Practical Low-light Image Enhancer. In *MM 2019 - Proceedings of the 27th ACM International Conference on Multimedia*, 1632–1640 (2019). 10.1145/3343031.3350926

[151] Wang, H., Xu, K. & Lau, R. W. H. Local Color Distributions Prior for Image Enhancement. (2022). https://hywang99.github.io/lcdpnet/

[152] Nguyen, H., Tran, D., Nguyen, K., Nguyen, R. & PSENet Progressive Self-Enhancement Network for Unsupervised Extreme-Light Image Enhancement. (2022). https://arxiv.org/abs/2210.00712

[153] Zamir, S. W. et al. Learning Enriched Features for Fast Image Restoration and Enhancement. (2022). https://arxiv.org/abs/2205.01649 10.1109/TPAMI.2022.3167175 35417348

[154] Tan, X. & Triggs, B. Enhanced local texture feature sets for face recognition under difficult lighting conditions. *IEEE Trans. Image Process.* **19**, 1635–1650. 10.1109/TIP.2010.2042645 (2010). 20172829 10.1109/TIP.2010.2042645

[155] Han, F., Yao, J., Zhu, H. & Wang, C. Underwater image processing and object detection based on deep CNN method. *J. Sensors* **2020**, 6707328. 10.1155/2020/6707328 (2020).

[156] Jo, W., Kim, S., Lee, C. & Shon, T. Packet preprocessing in CNN-based network intrusion detection system. *Electronics* **9**, 1151. 10.3390/ELECTRONICS9071151 (2020).

[157] Beeravolu, A. R. et al. Preprocessing of breast cancer images to create datasets for deep-CNN. *IEEE Access* **9**, 33438–33463. 10.1109/ACCESS.2021.3058773 (2021).

[158] Cui, Z. et al. You Only Need 90K Parameters to Adapt Light: A Light Weight Transformer for Image Enhancement and Exposure Correction. (2022). https://arxiv.org/abs/2205.14871

[159] Bai, J. et al. Retinex-based Mamba for Low-light Image Enhancement. (2024). https://arxiv.org/abs/2405.03349

[160] Guo, C. et al. Zero-Reference Deep Curve Estimation for Low-Light Image Enhancement. In *Proc. IEEE/CVF Conf. Computer Vision and Pattern Recognition (CVPR)*, 1780–1789 (2020).

[161] Nsampi, N. E., Hu, Z. & Wang, Q. Learning Exposure Correction Via Consistency Modeling. (2021). https://github.com/elientumba2019/Exposure-Correction-BMVC-2021

[162] Wang, T. et al. Ultra-High-Definition Low-Light Image Enhancement: A Benchmark and Transformer-Based Method. (2022). https://arxiv.org/abs/2212.11548

