# Supplementary material for: Enhancing life jacket detection for maritime search and rescue using YOLO models and illumination-robust preprocessing techniques
Source: Sci Rep. 2026 Jun 8;16:25997. doi: 10.1038/s41598-026-55717-0 (PMC13486517; doi:10.1038/s41598-026-55717-0)
Supplement: Supplementary file 1 [file 41598_2026_55717_MOESM1_ESM.docx]

**Supplementary Information for: Enhancing Life Jacket Detection for Maritime Search and Rescue Using YOLO Models and Illumination-Robust Preprocessing Techniques**

Kwong Jun Kit, Kwan Ban Hoe, Humaira Nisar, Tan Tian Swee, Hum Yan Chai

**Supplementary Table S1:** Pseudocode for Image Preprocessing Pipeline to Enhance Model Robustness Under Varying Lighting Conditions

**Supplementary Table S2:** Evaluation Workflow for YOLO Using Standard Object Detection Metrics

| **Step** | **Description** |
| --- | --- |
| 1 | Set working directory to Ultralytics root. |
| 2 | Define evaluation parameters: task = detect, mode = val, split = test, etc. |
| 3 | Load trained YOLO model weights (best.pt). |
| 4 | Load dataset using specified YAML configuration. |
| 5 | Extract the test split. |
| 6 | Run evaluation using defined parameters (image size, confidence, IoU, batch). |
| 7 | Compute and output: Precision, Recall, mAP@0.5, and mAP@0.5:0.95. |
| 8 | Return results for analysis and comparison. |

**Supplementary Table S3:** Inference Procedure for YOLO Model with Post-Processing and Result Visualization

| **Step** | **Description** |
| --- | --- |
| 1 | Import YOLO model class from the Ultralytics package. |
| 2 | Load trained YOLO model weights (best.pt). |
| 3 | Define input image path. |
| 4 | Run inference on the input image using the YOLO model. |
| 5 | For each detection result, extract: bounding boxes, masks, keypoints, etc. |
| 6 | Filter out low-confidence detections (threshold = 0.5). |
| 7 | Visualize the results on the image. |
| 8 | Save the annotated image to disk. |

**Supplementary Figure S1:** Comprehensive workflow for life jacket detection, encompassing data acquisition, annotation, dataset partitioning, model training with fixed parameters, and performance evaluation across detection models.

**Supplementary Figure S2:** Experimental workflow for evaluating life jacket detection models under varying lighting conditions using image enhancement techniques on overexposed and underexposed test sets.
